# A flexible GAS belt responds to pore mutations changing the ion selectivity of proton-gated channels

**DOI:** 10.1085/jgp.202112978

**Published:** 2021-11-12

**Authors:** Zhuyuan Chen, Sheng Lin, Tianze Xie, Jin-Ming Lin, Cecilia M. Canessa

**Affiliations:** 1 Department of Basic Sciences, Tsinghua University School of Medicine, Beijing, China; 2 Department of Chemistry, Beijing Key Laboratory of Microanalytical Methods and Instrumentation, Ministry of Education Key Laboratory of Bioorganic Phosphorus Chemistry & Chemical Biology, Tsinghua University, Beijing, China; 3 Cellular and Molecular Physiology, Yale University, New Haven, CT

## Abstract

Proton-gated ion channels conduct mainly Na^+^ to induce postsynaptic membrane depolarization. Finding the determinants of ion selectivity requires knowledge of the pore structure in the open conformation, but such information is not yet available. Here, the open conformation of the hASIC1a channel was computationally modeled, and functional effects of pore mutations were analyzed in light of the predicted structures. The open pore structure shows two constrictions of similar diameter formed by the backbone of the GAS belt and, right beneath it, by the side chains of H28 from the reentrant loop. Models of nonselective mutant channels, but not those that maintain ion selectivity, predict enlargement of the GAS belt, suggesting that this motif is quite flexible and that the loss of stabilizing interactions in the central pore leads to changes in size/shape of the belt. Our results are consistent with the “close-fit” mechanism governing selectivity of hASIC1a, wherein the backbone of the GAS substitutes at least part of the hydration shell of a permeant ion to enable crossing the pore constriction.

## Teaser

Chen et al. computationally model the open conformation of the hASIC1a channel to predict that mutations that eliminate channel selectivity cause a widening of the GAS belt. Their findings implicate the GAS belt diameter in channel kinetics and ion selectivity.

## Introduction

Acid-sensing ion channels (ASICs) are proton-activated cation channels broadly expressed in neurons. They are members of the degenerin–epithelial Na^+^ channel family (DEG-ENaC; Hanukoglu and Hanukoglu, 2016), which constitutes a large group of metazoan ion channels that are closely related by sharing a common structure but exhibit different means of activation, kinetics, and selectivity of Na^+^ over K^+^ and other cations (García-Añoveros et al., 1997; Price et al., 1996; Waldmann et al., 1996). The isoform ASIC1a participates in diverse physiological functions such as modulation of synaptic transmission (González-Inchauspe et al., 2017; Kreple et al., 2014; Wemmie et al., 2002), fear sensing (Du et al., 2017; Taugher et al., 2017), addiction-related behaviors (Kreple et al., 2014), and nociception (Bohlen et al., 2011; Deval and Lingueglia, 2015). It is also implicated in pathological processes such as ischemic stroke (Duan et al., 2011; Xiong et al., 2004), epilepsy (Ali et al., 2006; Luszczki et al., 2009; Lv et al., 2011; N’Gouemo, 2008; Ziemann et al., 2008), and multiple sclerosis (Arun et al., 2013; Bernardinelli et al., 2007; Friese et al., 2007; Vergo et al., 2011).

ASICs are made of three pore-forming subunits, and each subunit has a large extracellular domain (ECD), two transmembrane (TM) helices, and a short intracellular N-terminus and C-terminus. Although it has been known that mutations in a conserved segment of the N-terminus proximal to TM1 alter ion selectivity and kinetics (Coscoy et al., 1999; Pfister et al., 2006; Sheikh et al., 2021), the lack of structural information of this domain has made it difficult to explain the mechanism(s) underlying the functional changes induced by those mutations. A genetic form of human pseudohypoaldosteronism produced by a mutation of the glycine residue in the His-Gly (HG) motif of the β-subunit of ENaC reduces activity of the channel, most likely by decreasing the open probability (Chang et al., 1996; Gründer et al., 1997, 1999). In the roundworm *Caenorhabditis elegans*, mutations of the same motif in DEG channels lead to the loss of light touch (Tavernarakis et al, 2001). Several mutations in the pre-TM1 segment of rat ASIC2 reduce Na^+^ over K^+^ selectivity (Coscoy et al., 1999), and mutations in the same segment also decrease currents in ASIC1a (Pfister et al., 2006).

Recently, cryo-EM structures of chicken ASIC1 (cASIC1) extracted from native membranes using styrene maleic acid copolymer revealed that the N-terminus (V17^cASIC1^-L40^cASIC1^) of cASIC1 forms a loop that enters the lower part of the pore at pH 8.0 (Protein Data Bank [PDB] accession no. 6VTL) and pH 7.0 (PDB accession no. 6VTK), corresponding to the resting and steady-state desensitized states, respectively (Yoder and Gouaux, 2020). The reentrant loop has two short helical segments, Re-1 (residues I19–S25) and Re-2 (residues I31–F35), connected by a loop that contains the highly conserved HG motif (Chang et al., 1996). Of note, the His side chain from the HG motif of each of the three subunits together form the smallest constriction of the ion pathway in both the closed and desensitized conformations, suggesting that this constriction may constitute the selectivity filter of ASICs and, by extension, other members of the DEG-ENaC family.

Previously, however, another candidate for the selectivity filter was proposed based on mutagenesis results of three conserved residues located midway in TM2 of the subunits of ENaC (α, β, and γ) that, when mutated, alter ion selectivity (Kellenberger et al., 2001, 1999a, 1999b; Kellenberger and Schild, 2002; Yang and Palmer, 2018). The equivalent residues, Gly-Ala-Ser (GAS) in ASIC, mostly abolish currents (Carattino and Della Vecchia, 2012; Li et al., 2011; Lynagh et al., 2020), but more subtle changes introduced in the backbone of GAS by unnatural amino acids produced measurable changes in selectivity (Lynagh et al., 2017). Subsequently, the crystal structure of the open channel conformation of the cASIC1–MitTx1 complex revealed that GAS forms an unwound α-helix that enables swapping of the lower half of TM2 (TM2b) with the adjacent subunit. The stretch configuration of these three residues was dubbed the “GAS belt” (Baconguis et al., 2014). Significantly, in the open pore structure of the cASIC1–MitTx1 complex, which does not include the reentrant loop, the GAS belt is the narrowest segment of the ion pathway, giving additional support to the possibility that it forms the selectivity filter of ASICs.

Recently, molecular dynamics free energy simulations identified high electrostatic negative potential in the lower pore at the sites corresponding to E452 and D455 in TM2b, raising the possibility that those two negatively charged residues may be critical for discrimination between Na^+^ and K^+^ in the ion pathway, therefore also representing potential candidates for a selectivity filter (Kasimova et al., 2020; Lynagh et al., 2020, 2017).

In this study, we have taken advantage of the recently revealed N-terminal reentrant loop structure of cASIC1 in the closed/desensitized states to predict models of hASIC1a in the open and closed/desensitized conformations by homology modeling followed by minimization with RosettaMembrane energy function. Combining electrophysiology data and predicted lower pore state-dependent structures, we were able to identify distinct interaction networks between the reentrant loop and the two TM domains that serve to stabilize the pore in open or closed/desensitized states. We show that mutations that disrupt interaction networks between the GAS belt and the reentrant loop change kinetics and ion selectivity. Notably, predicted structures of nonselective mutant channels show widening of the GAS but not the predicted structure of selective mutants, suggesting that the GAS belt diameter is not rigid, but it widens in response to mutations in the lower pore that lead to loss of ion selectivity.

## Materials and methods

### Modeling of hASIC1a in the resting and desensitized conformations

Structural models of hASIC1a (residues V16–H463; UniProt accession no. P78348) in the closed and desensitized states were developed by homology modeling using the protein structure prediction software Rosetta (version 3.12; Leman et al., 2020). The structure of cASIC1 (UniProt accession no. Q1XA76) from cryo-EM determined at high pH (PDB accession no. 6VTL; Yoder and Gouaux, 2020) served as a template for the closed state model. The structure of cASIC1 determined at low pH (PDB accession no. 6VTK; Yoder and Gouaux, 2020) was used for modeling the desensitized state structure. Resting or desensitized hASIC1a models were built by threading their amino acid sequences through the structures of cASIC1 under the RosettaCM (comparative modeling with Rosetta) protocol and guided by pairwise amino acid sequence alignments created with ClustalW (Larkin et al., 2007; Song et al., 2013). The resting or desensitized hASIC1a model with the highest score was further refined by minimization in Cartesian space with RosettaMembrane energy function (Barth et al., 2007) while applying weak harmonic restraints to the positions of all backbone heavy atoms in PDB accession no. 6VTL or 6VTK, respectively. Amino acid side chain positions were refined by a simulated annealing search algorithm, referred to as rotamer packing in Rosetta. The resting or desensitized hASIC1a model with the highest score was selected, and C3 symmetry of the trimeric hASIC1a structure was enforced according to symmetry definition files described by DiMaio et al. (2011). The resting or desensitized hASIC1a model with the highest score was chosen as the final model. A minimum of 100 models were developed for each step, and the quality of all highest-score models in each step was verified by MolProbity analysis (Davis et al., 2007).

### Modeling of hASIC1a in the open conformation

For modeling of hASIC1a in the open conformation, the structure of cASIC1 in complex with the MitTx at pH 5.5 (PDB accession no. 4NTW; Baconguis et al., 2014) was used as a template for modeling the ECD and TM domains of hASIC1a. Prior to homology modeling, missing loop residues in the open state structure of cASIC1 (D297, S298) were added with RosettaRemodel (Huang et al., 2011) guided by PSIPRED (Jones, 1999) secondary structure prediction. Then this amended cASIC1 structure was used as a template to guide the modeling of hASIC1a in the open conformation by the RosettaCM protocol and sequence alignments (Larkin et al., 2007; Song et al., 2013). After modeling the ECD and TM domains of hASIC1a in the open conformation, the model with the highest score was selected, and N- and C-terminal structures were extended to include the pre-TM1 reentrant loop and distal TM2 residues by the built-in multiple input templates function in RosettaCM. Corresponding pre-TM1 reentrant loop (V17–V45) and distal TM2 (A456–H462) structures presented in PDB accession nos. 6VTL and 6VTK (cASIC1) were used as templates for the multiple input templates function, while the ECD and TM domain structures of the homology modeled hASIC1a open state structure were kept unchanged under strong restraints (Song et al., 2013). The extended hASIC1a model (V16–H463) with the highest score was subsequently refined by minimization in Cartesian space with the RosettaMembrane energy function (Barth et al., 2007) while applying weak harmonic restraints to the positions of all backbone heavy atoms in the amended 4NTW template to relax this completed hASIC1a model in the open conformation. Amino acid side chain positions were refined by a simulated annealing search algorithm, referred to as rotamer packing in Rosetta. The model with the highest score was chosen, and C3 symmetry of the trimeric hASIC1a structure was enforced according to symmetry definition files described by DiMaio et al. (2011). The model with the highest score was selected as the final model. A minimum of 100 models were developed for each step, and the quality of all highest-score models in each step was verified by MolProbity analysis (Davis et al., 2007). Rosetta commands and scripts used to generate hASIC1 channel models are shown in Data S3.

### Protein stability prediction for hASIC1a mutants

Free energy changes of hASIC1a structures owing to amino acid mutations were calculated with the Rosetta Cartesian ddG protocol (Park et al., 2016) and the RosettaMembrane all-atom energy function (Barth et al., 2007). In short, the Cartesian ddG protocol models mutation-induced conformational and energetic changes by a series of “backrub” moves (15,000 steps in this study) of the protein backbone coupled to side chain repacking in an 8 Å shell around the mutation site, followed by minimization of all protein backbone and side chain degrees of freedom. This sampling method aims at improving the prediction for mutations with a large change in amino acid side chain volume. Afterward, structures were minimized in Cartesian space until convergence was reached and the change of the Rosetta score between subsequent iterations was <1.0 Rosetta energy unit (REU). To avoid large deviations from the input structure, Cα atom pair distance restraints with a harmonic penalty function were applied during minimization. A mutation was modeled in each of the three hASIC1a subunits simultaneously by enforcing C3 symmetry according to Rosetta symmetry definition files. 10 separate Cartesian ddG trajectories were conducted for each mutant and WT hASIC1a, and the Rosetta energy change (ΔΔG) was calculated as average score difference between the 10 top-scoring mutant and WT structures. Stability calculations were repeated at least 10 times for each mutant, and the mean ΔΔG was used for making stability predictions.

### Modeling of hASIC1a mutants in the open conformation

For modeling of hASIC1a mutants in the open conformation, the structure with the score closest to the mean score in the ΔΔG set for each mutant was subsequently refined by minimization in Cartesian space with the RosettaMembrane energy function (Barth et al., 2007). Amino acid side chain positions were refined by a simulated annealing search algorithm, referred to as rotamer packing in Rosetta. The model with the highest score was chosen, and C3 symmetry of the trimeric hASIC1a structure was enforced according to symmetry definition files described by DiMaio et al. (2011). The model with the highest score was selected as the final model. A minimum of 100 models were developed for each step, and the quality of all of the highest-score models in each step were verified by MolProbity analysis (Davis et al., 2007).

### Identification of amino acid interactions in modeled structures

Hydrogen bonding was identified by a built-in PyMOL function: action → find → any contacts → between chains within 4.0 Å (with actual interacting distance between heavy atoms shown in PyMOL). Pi–Pi interactions were also identified by built-in PyMOL function: action → find → Pi interactions → all.

### cDNA constructs and mutagenesis

Plasmid pCDNA3.1(−) containing hASIC1-hemagglutinin (HA) served as a template for introducing point mutations using the QuikChange system according to the manufacturer’s instructions. The correct DNA sequence of all constructs was verified by sequencing.

### In vitro synthesis of cRNAs and injection of *Xenopus laevis* oocytes

pcDNA3.1 plasmids were linearized with HindIII followed by in vitro synthesis of RNA using mMESSAGEmMACHINE T7 according to the manufacturer’s instructions. The concentration of RNA was adjusted to 300 ng/µl using a NanoDrop device. 5 ng cRNA solution were injected per oocyte, followed by incubation at 18°C for 18–24 h before experiments. Oocytes were harvested from female *Xenopus* according to a protocol approved by the institutional animal care and use committee of Tsinghua University (protocol 07749). The Association for Assessment and Accreditation of Laboratory Animal Care International has accredited the Tsinghua amphibian animal facility. Harvested oocytes were treated with 20 mg/10 ml of collagenase for 30–40 min in amphibian isotonic buffer without Ca^2+^ (in mM: 88 NaCl, 1 KCl, 2.4 NaHCO_3_, 0.82 MgSO_4_, and 10 HEPES-NaOH, pH 7.6) at room temperature. Cells were extensively washed, injected with cRNA, and incubated in the same buffer supplemented with 0.41 mM CaCl_2_ and 0.33 mM Ca(NO_3_)_2_ for 18–24 h at 18°C.

### Two-electrode voltage clamp (TEVC)

Whole-cell currents were measured using a TEVC (Oocyte-Clamp OC-725C; Warner Instrument Corp.) with PowerLab 8/35 (ADInstruments) running LabChartPro software. Cells were placed in a fast exchange perfusion chamber with high flow delivered by gravity. Perfusion solutions had the following composition (in mM): 100 NaCl, 4 KCl, 1.5 CaCl_2_, 10 HEPES, and 10 Mes, and pH was adjusted to the desired values with NMDG. Pipette resistances were 0.5–1 MΩ when filled with 3 M KCl. Oocytes were voltage clamped at −60 mV. ASIC currents were activated by changing the external solution from a conditioning pH of 7.4 (or any other indicated value) to a more acidic test pH for 10–40 s until currents completely desensitized or the sustained current reached a plateau. The external solution was returned to pH 7.4 or changed to the indicated preconditioning pH for 60 s. The apparent pH of activation (pH_50a_) is the pH value in the range of 7.2 to 6.0 that induces half-maximal current.

### Normalization of current amplitudes

Measurements of peak currents of mutants were compared with currents elicited in cells expressing WT hASIC1a from the same batch of oocytes activated by pH 6.0: I_peak_(mutant)/I_mean_(WT). Measurements of reversal potential and calculation of permeability ratios were conducted using a ramp protocol consisting of a linear change in voltage from −80 mV to +60 mV of 200-ms duration. Ramps were applied with channels closed (pH 7.4) and right after activation (pH 6.0), when currents reach a maximal value. The composition of the solutions during the two ramps was identical except for the pH values: 100 mM NaCl or KCl or CsCl, 5 mM HEPES, 5 mM Mes, and 2 mM CaCl_2_. Subtraction of currents obtained with pH 7.4 and pH 6.0 was used to calculate the reversal potential for each cation. The subtraction procedure served to isolate the proton-induced component from leaks. Permeability ratios were calculated from the shift of the corrected reversal potential ΔVrev of the I-V relationship according to the following function:

$$\frac{P_{x}}{P_{Na}}=\exp(F*\frac{\Delta V_{rev}}{RT})$$

V_rev_ of Na^+^, K^+^, and Cs^+^ was measured and calculated in the same oocyte, and at least three oocytes were measured for each group (hASIC1a WT and mutants). For mutants with small peak currents and low recovery from desensitization (e.g., H28R, E452Q, D455S/H/T/N, and E459F), the reversal potential of Na^+^, K^+^, or Cs^+^ was calculated only from cells with proton-activated currents ≥3 µA/cell, sometimes requiring use of different oocytes for each cation.

### Kinetics of desensitization

The decay of peak currents elicited by pH 6.0 was fit to a single exponential function: a*exp(−t/τ_d_), where a is the peak current value, t is the time in seconds, and τ_d_ is the time constant of desensitization (s).

### Recovery from desensitization

Channels were activated with pH 6.0 until the current returned to 0 or stabilized at a plateau level. The perfusion solution was changed to pH 7.4 for 30 s, and a second activation (pH 6.0) was applied. The recovery ratio (in percent) was calculated as the fraction of a second over the first stimulus peak current. If no current was elicited by pH 6.0, the measurement was repeated at pH 5.0.

### Calculation of pH_50a_

The concentration response curves were fit to the Hill function: I = 1/(1 + [10^pH50a^/10^pH^]^n^), where pH_50a_ is the pH at which the half-maximal activation/desensitization of the maximal current was achieved and *n* is the Hill coefficient. All data are presented as mean ± SD. They represent the mean of 5–10 individual measurements in different oocytes and at least 2 different batches of oocytes.

### Patch clamping

Currents were recorded from excised patches in the outside-out configuration using a HEKA patch-clamp EPC10 amplifier and PATCHMASTER acquisition software version 2x90.2 (HEKA Electronik). The pipette solution contained (in mM): 100 KCl and 20 HEPES, pH 7.4. The bath solution contained (in mM): 100 NaCl, 4 KCl, 2 CaCl_2_, and 20 HEPES/Mes, adjusted to pH 7.4 or lower pH with NMDG. The membrane potential was held at −60 mV. Patches were perfused with a solution of pH 7.4 to establish the baseline current, followed by activation with low-pH solutions (pH range of 7.1–6.5 for activation) using a fast-exchange perfusion system (SF-77B Perfusion Fast-Step System; Warner Instruments). Experiments were conducted at room temperature.

### Surface biotinylation

Cells cultured on 24-well plates were transfected with 1 µg DNA per well using Lipofectamine 2000 (Invitrogen). Cells were harvested after 24 h and washed once with PBS without Ca/Mg (PBS). Cells were biotinylated with 2 mM EZ-Link Sulfo-NHS-LC-Biotin (Thermo Fisher Scientific) on ice for 2 h before they were washed three times with PBS plus 100 mM glycine for quenching of biotinylation. Cells were lysed with TENT (in mM: 50 Tris [pH 7.4], 5 EDTA, 150 NaCl, and 1% [wt/vol] Triton X-100) supplemented with a cocktail of protease inhibitors (Roche) on ice for 30 min, followed by centrifugation at 10,000 rpm for 5 min at 4°C, and the supernatant was recovered. NeutraAvidin resins were then added to the supernatant to capture the biotinylated protein for 1 h on a rotator at room temperature. Biotinylated proteins bound to the beads were eluted with protein loading buffer after three washes with TENT. Before loading, the samples were heated for 10 min at 95°C.

### Immunoblot

HEK293T cells expressing the protein of interest cultured in a 24-well plate were harvested ∼24 h after transfection. Cells were washed once with PBS and lysed with TENT buffer (200 µl/well) supplemented with protease inhibitor cocktail (Roche) for 30 min on ice. The cell lysate was centrifuged at 10,000 *g* for 5 min at 4°C, and the supernatant (whole-cell lysate [WCL]) was recovered. 20 µl of WCL with 4 µl of 6× loading buffer were heated for 10 min at 95°C before being loaded onto gels. Samples were then run on 10% SDS-PAGE gels and transferred onto Immobilon-P 0.45-µm polyvinylidene difluoride membrane (Immobilon-P, IPVH00010; EMD Millipore; Bio-Rad Western blotting system). The blots were blocked with 5% (wt/vol) milk in TBST buffer (in mM: 137 NaCl, 20 Tris [pH 7.6], and 0.1% [wt/vol] Tween 20) for 1 h at room temperature. Blots were then incubated with the primary anti–HA-horseradish peroxidase (HRP; H6533-1VL; Sigma-Aldrich) at 1:1,000 dilution and anti–GAPDH-HRP (G9295-200UL; Sigma-Aldrich) at 1:5,000 dilution for WCL blots. Antibodies were diluted with 1% (wt/vol) milk in TBST buffer for 1 h at room temperature followed by three washes with TBST buffer at each step. Densitometric analyses of the Western blotting images (ChemiDoc MP Imaging System; Bio-Rad Laboratories) were performed using ImageJ software. Representative blots were presented together with mean ± SD values.

### Statistical analysis

Student’s *t* test was used for comparison of the mean of two groups. A statistically significant P value was set at 0.01 or less. When more than two groups were compared, one-way ANOVA was used. If a significant F value was calculated, it was followed by a post hoc Dunnett’s test for multiple comparisons, where the control group was WT hASIC1a. Mutants with eight or more data points were examined for constant variance (ratio of the largest and smallest SD <2), and for normal distribution (QQ plot). Comparison significance at the 0.01 level was chosen. Calculations were conducted with KaleidaGraph version 4.5.2 (Synergy Software). Statistical analysis results are included in Data S1 and Data S2.

### Online supplemental material

**Figure S1.**
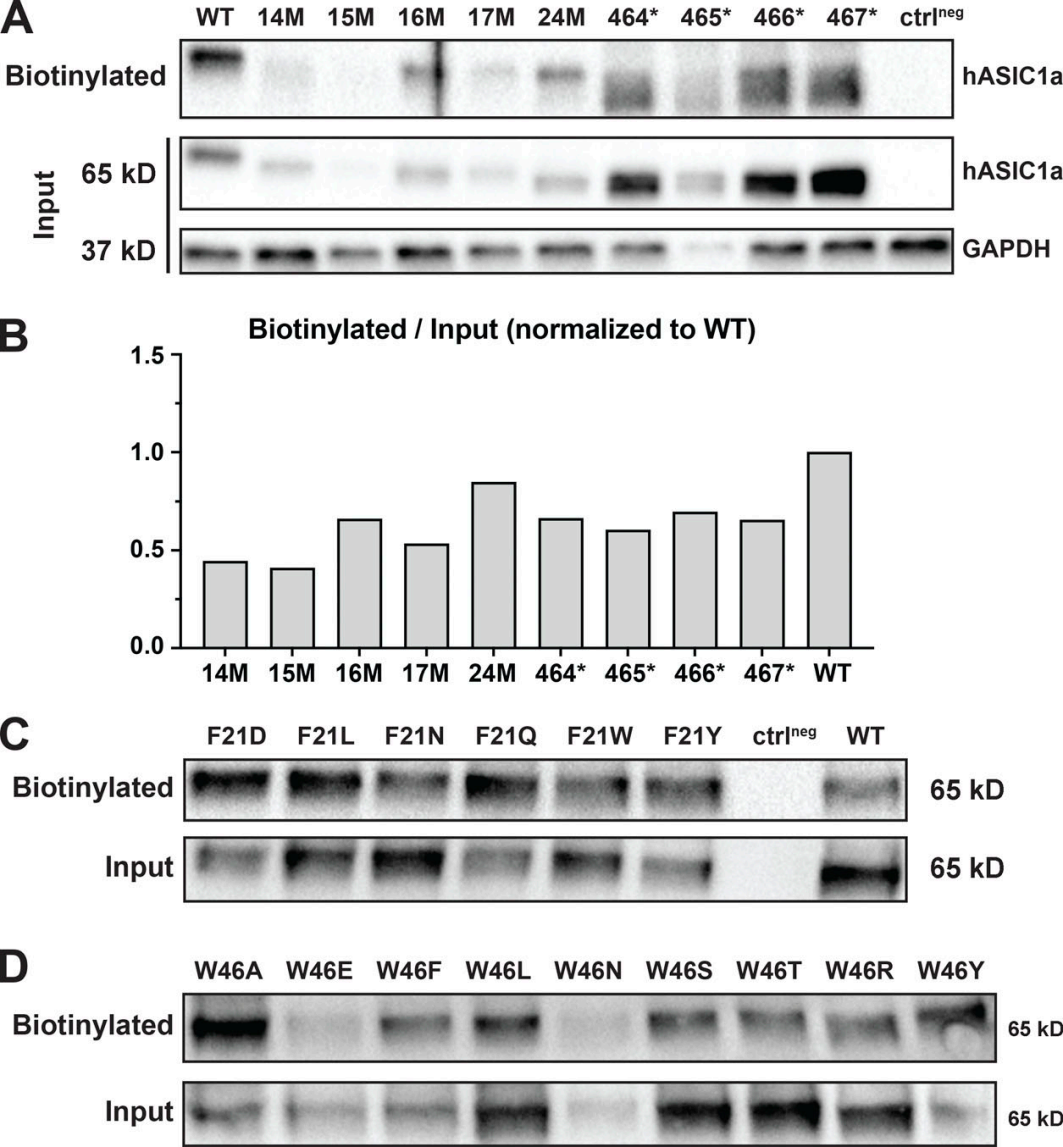
**Surface expression of nonfunctional hASIC1a mutants.** **(A)** Immunoblots of surface biotinylated HA WT and mutants with N- or C-terminal truncation at the indicated positions, total hASIC1a protein, and GAPDH protein in WCLs. The ctrl^neg^ sample is collected from nontransfected HEK293T cells; GAPDH is used as a loading control. Blots were incubated with anti–HA-HRP or anti–GAPDH-HRP antibody as indicated on the right. **(B)** Quantitative analysis of the immunoblots. Density values of the bands were measured using the ImageJ program. The ratio of the surface portion versus total amount in the WCL normalized to WT hASIC1a is shown as bars. **(C)** Immunoblots of surface expression of F21 hASIC1a mutants (biotinylated) compared to total lysate. **(D)** Similar experiment of W46 mutants.

**Figure S2.**
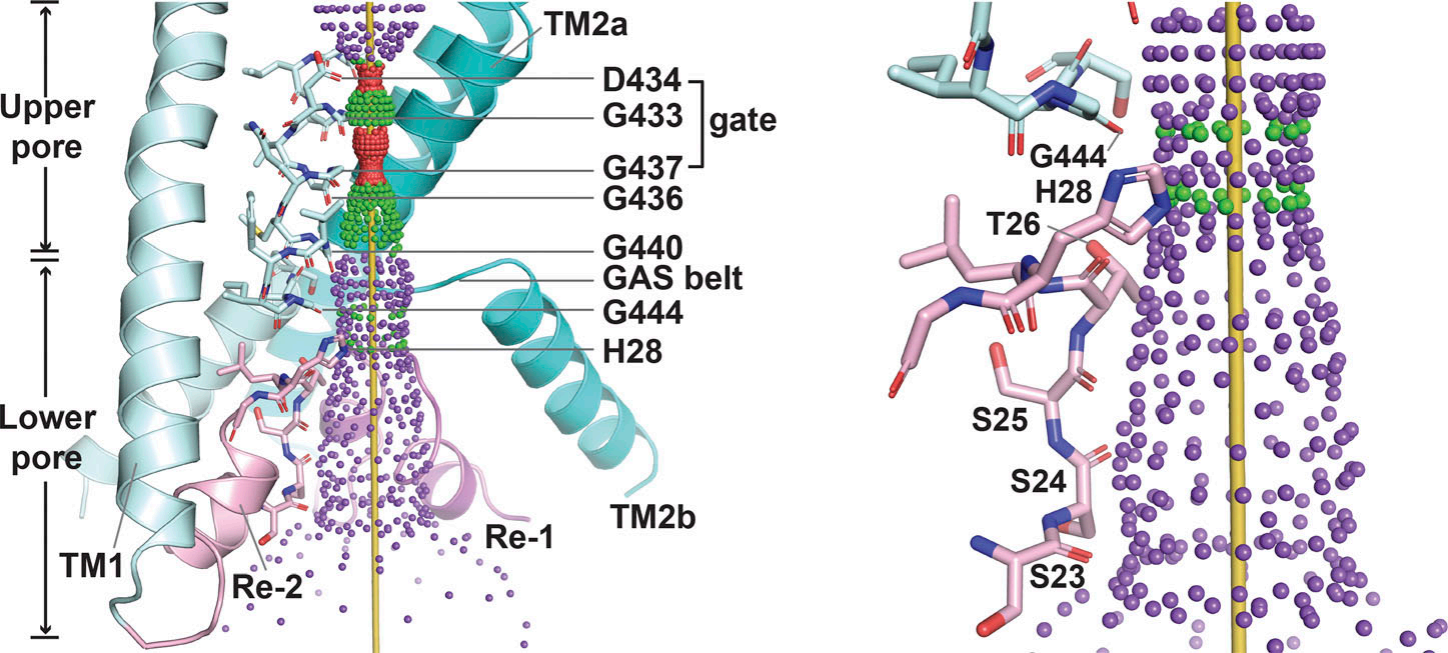
**Predicted pore structure of hASIC1a in the closed conformation.** Side view of the ion permeation pathway in the predicted resting conformation of hASIC1a (left panel). Only two subunits are shown for clarity. The TM domains are colored teal, and the reentrant loops are pink. A zoomed-in side view of the ion permeation pathway is shown with the following residues lining the permeation pathway: G444, H28, T26, S25, S24, and S23 (right panel).

**Figure S3.**
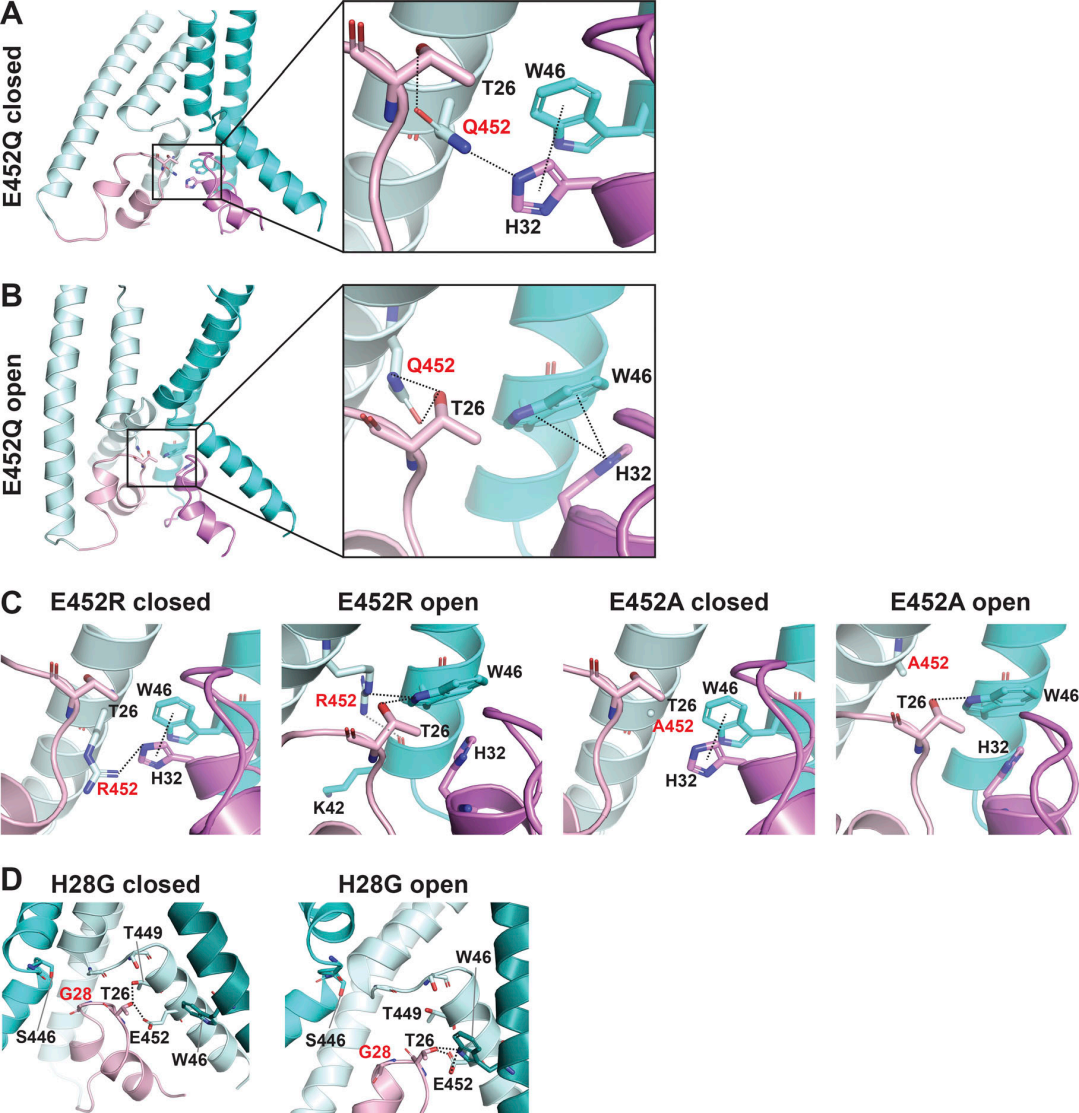
**The substitution E452Q loses a H-bond interaction with W46 in the predicted open conformation of Q452^hASIC1a^.** **(A and B)** Side view of the predicted closed (A) and open (B) conformations of E452Q, highlighting altered interaction networks (compare with Fig. 5 A) formed by residues Q452, T26, W46, and H32. **(C)** R452 retains interactions with H32 in closed and with W46 in open states enabling small currents. A452, which does not interact with any residue, is not functional. **(D)** G28 does not make a stabilizing contact with the GAS belt in closed or open conformations resulting in complete loss of function.

**Figure S4.**
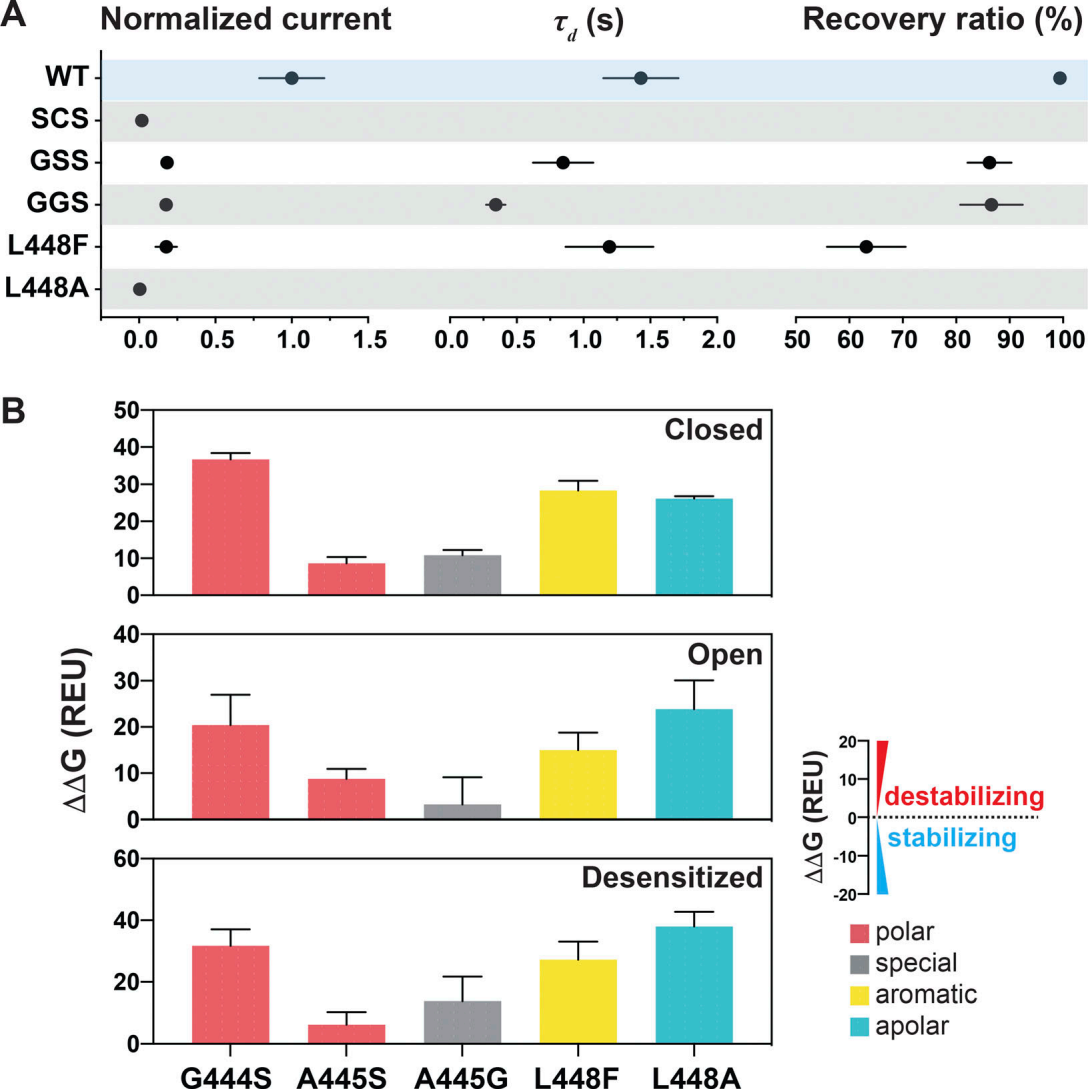
**Substitutions at the GAS or L448 alter channel kinetics by destabilizing the three states.** **(A)** Summaries of normalized current, *τ*_d_, and recovery ratio for GAS and L448 substitutions. Data are presented as mean ± SD of three independent experiments for at least five *Xenopus* oocytes (*n* = 5–10) for each construct tested. All oocytes were incubated at least 30 s in preconditioning buffer (pH 7.4) for channel recovery before the next activation. Recovery ratio (expressed in percent) was calculated as I_acti(n+1)_/I_acti(n)_. Individual values are shown in Data S2. **(B)** Free energy change (ΔΔG) calculations of the above substitutions in the closed, open, and desensitized states compared with the WT hASIC1a in the corresponding conformations. A ΔΔG value > 1 is considered to be destabilizing, while a ΔΔG value less than −1 is considered to be stabilizing. All values are presented as mean ± SD. 10 runs of free energy calculations were conducted in replicates for each mutant and the WT hASIC1; 10 free energy values of the WT and all mutants were sorted from smallest to largest before free energy values of the WT were subtracted from those of each mutant (10 ΔΔG values obtained for each mutant). Individual values are provided in Data S2.

**Figure S5.**
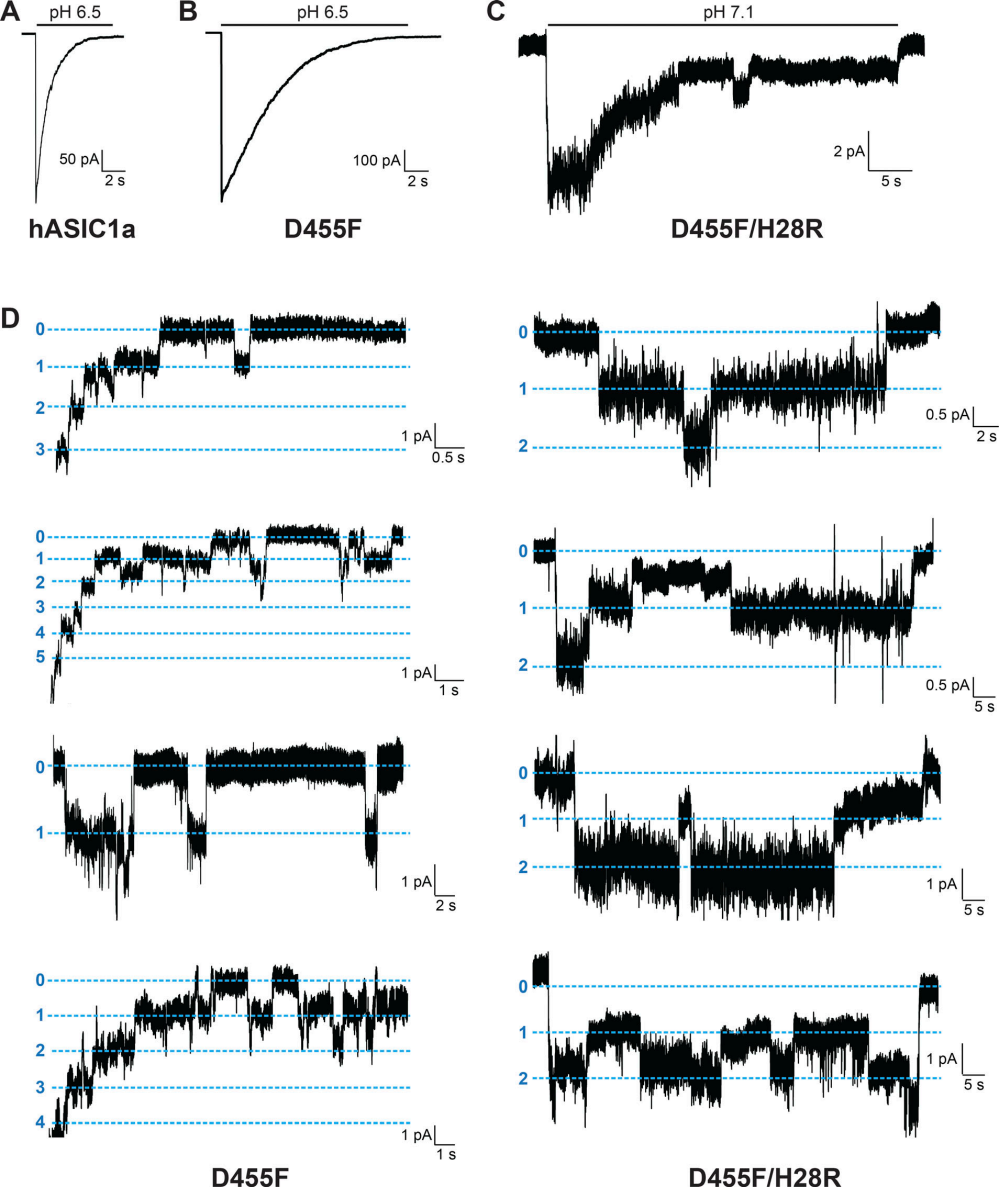
**D455F and D455F/H28R channels exhibit long single-channel opening events.** **(A)** Representative examples of excised outside-out membrane patches containing a large number of WT hASIC1a channels activated by external pH 6.5. **(B)** hASIC1a with D455F substitution. **(C)** Representative example of an excised outside-out membrane patch containing four or five channels of hASIC1a with two substitutions D455F/H28R activated by external pH 7.1. **(D)** Representative examples of excised outside-out membrane patches containing multiple (D455F; left panels) or one to three (D455F/H28R; right panels) channels. Holding potential was −60 mV. External solution was 100 mM Na^+^. Pipette solution was 100 mM K^+^. Dashed blue lines indicate current levels when 0, 1, 2, 3, or 4 channels were open.

**Figure S6.**
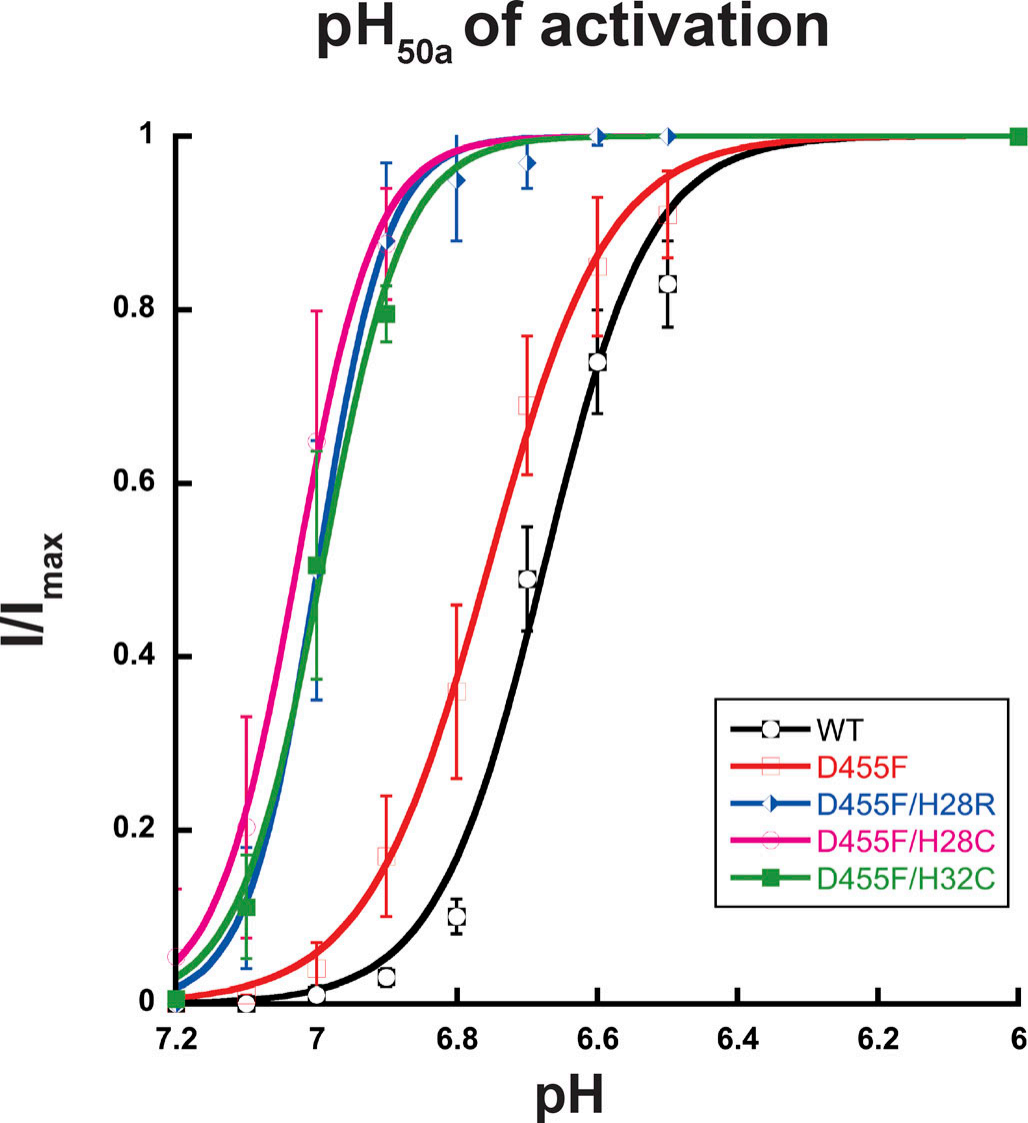
**D455F and D455F double mutants exhibit high apparent proton affinity for activation.** Increasing proton concentration response curves of activation. The pH_50a_ values were 6.76 for D455F (red curve), 7.00 for D455F/H28R (blue curve), 7.03 for D455F/H28C (pink curve), and 6.99 for D455F/H32C (green curve) versus 6.68 for the WT hASIC1a (black curve). Lines are the fit to the Hill function. Each data point represents three to six independent measurements.

**Figure S7.**
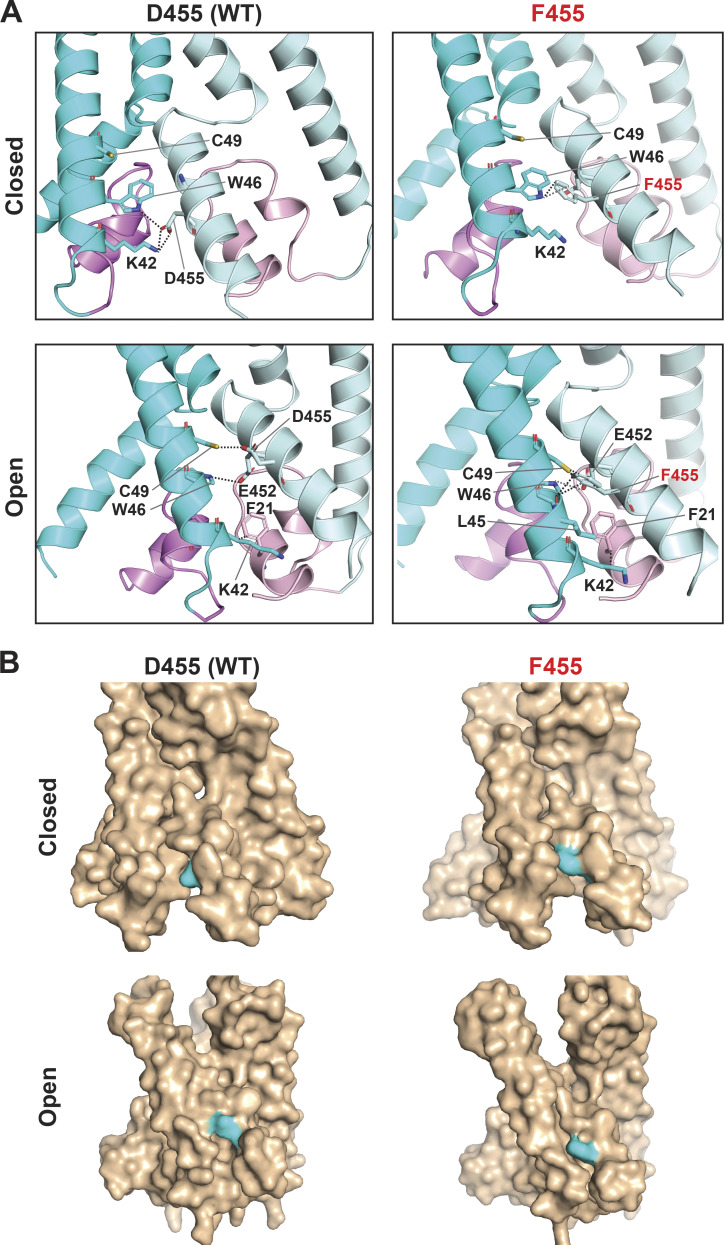
**Potential interactions of D455 and F455 with TM1 and with membrane lipids. (A)** Side view of the predicted WT hASIC1a and mutant F455 in the resting (upper panel) and open (lower panel) conformations highlighting interactions formed by residues D/F455. Only two subunits at the membrane level are shown. **(B)** Above structures shown on surface representation. The side chains of residues D455 and F455 are in contact with the membrane lipids shown by the exposed surface colored in cyan.

Fig. S1 shows the surface expression of WT and nonfunctional hASIC1a mutants. Fig. S2 shows the predicted pore structure of hASIC1a in the closed conformation. Fig. S3 shows the predicted changes in the interaction network of the mutant E452Q in closed and open conformations. Fig. S4 shows that substitutions at the GAS or L448 alter channel kinetics by destabilizing the three states. Fig. S5 shows the single-channel recordings of D455F^hASIC1a^ and D455F/H28R^hASIC1a^. Fig. S6 shows the apparent proton affinity pH_50a_ of D455F and D455F double mutants. Fig. S7 shows the potential interactions of D455 and F455 with TM1 and with membrane lipids. Data S1 is a data file of ΔΔG. Data S2 is a data file of all electrophysiological measurements shown in the figures. Data S3 lists Rosetta commands and scripts used to obtain the predicted open, closed, and desensitized structures of hASIC1a.

## Results

### N-terminal sequences proximal to TM1 and distal segment of TM2 are required for hASIC1a function

**Figure 1.**
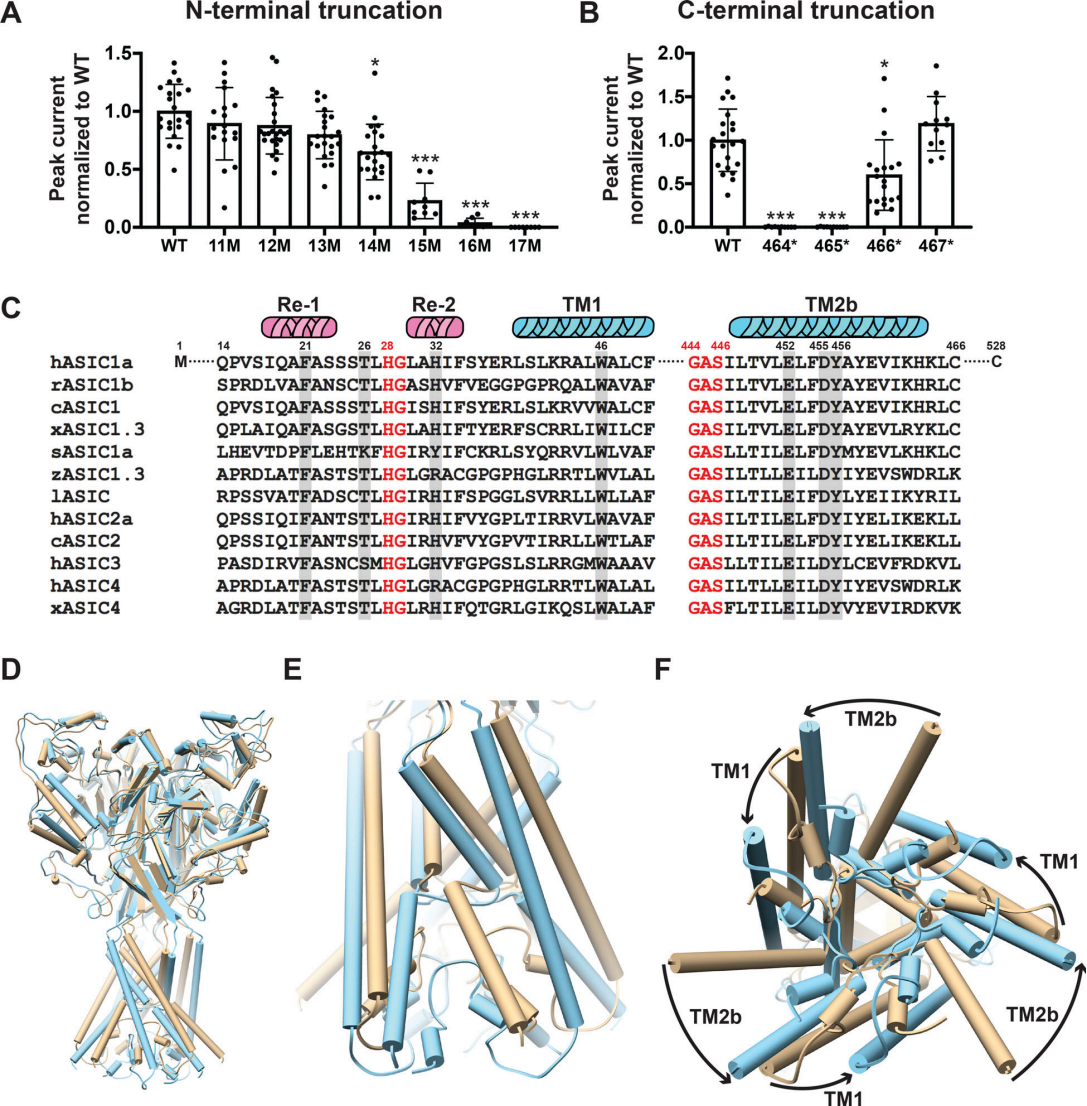
**Essential N- and C-terminal residues for hASIC1a function. (A)** Normalized peak currents of indicated hASIC1a N-terminal truncations measured from *Xenopus* oocytes injected with cRNA of WT hASIC1a or with truncations examined by TEVC. Normalized peak currents are presented as mean ± SD of three independent experiments of at least 10 oocytes (*n* = 10–25) for each construct. One-way ANOVA post hoc Dunnett’s F test 49.2; ***, P < 0.0001. Individual measurements are shown in Data S2 for A and B. **(B)** Normalized peak currents of indicated hASIC1a C-terminal truncations measured and analyzed as in A. ***, P < 0.0001; *, P = 0.01. **(C)** Sequence alignment of amino acids encoding the lower pore of ASICs. From various species using Lasergene DNASTAR-MegAlign software. Highlighted residues were examined in this work. c, chicken; h, human; l, lamprey; r, rat; s, shark; x, *Xenopus*; z, zebrafish. **(D–F)** Pore opening repositions functionally essential residues in the lower pore of hASIC1a. **(D)** Side view of the predicted hASIC1a in the open conformation (blue) superimposed onto the predicted hASIC1a in the resting conformation (tan). **(E)** Enlarged view of the TM domains and the reentrant loop. **(F)** View of the TM domains from the intracellular side shows rotation of TM1 and TM2 around the threefold axis and the predicted repositioning of the reentrant loop from the closed (tan) to the open (blue) conformation.

Cryo-EM structures of the proximal N-terminus from V17 and distal TM2 extending to H462 of cASIC1 have recently been resolved in both resting and steady-state desensitized conformations (Yoder and Gouaux, 2020). We first determined the minimal number of residues that are essential to maintain normal hASIC1a function. Sequential deletions of the N-terminus were made from positions 11 to 17 and of the C-terminus by introducing stop codons from positions K464 to R467. Oocytes injected with each of the above deletions were examined with TEVC. Truncations from positions 14 to 16 progressively reduced currents, and position 17 was nonfunctional (Fig. 1 A). The majority of the C-terminus from R467 was removed without impairing channel function (Fig. 1 B). Protein expression levels and traffic to the plasma membrane were not affected by deletions, as indicated by quantification of the total protein and surface biotinylated fraction (Fig. S1, A and B) The results indicate that the minimal sequence required for normal channel function extends from residues 14 to 466 of hASIC1a. Therefore, the most recent cryo-EM structure of cASIC1 misses two crucial residues (Q15, P16) and does not show the side chains of V17 and S18 in the N-terminus. The distal TM2b does not show the side chain of H462 and R463 to C465, which are also essential for function (Yoder and Gouaux, 2020).

### Predicted structure of the lower open pore architecture of hASIC1a

To understand the functional properties of the hASIC1a pore, it is necessary to know the structure of the pore in closed and open conformations. Because currently only the closed/desensitized structures with the reentrant loop are available from cASIC1, we modeled the structure of hASIC1a in closed, open, and desensitized states, all of them with the reentrant loop as described in Materials and methods. In short, we used the cASIC1 resting state structure as a template (PDB accession no. 6VTL; Yoder and Gouaux, 2020) to obtain the hASIC1a resting state structure. Similarly, cASIC1 in the desensitized state served as a template for desensitized hASIC1a (PDB accession no. 6VTK; Yoder and Gouaux, 2020). To model hASIC1a in the open conformation, we used the cASIC1 open state structure as a template (PDB accession no. 4NTW; Baconguis et al., 2014), but because this cASIC1 structure does not include the proximal N- and C-termini, we used proximal N- and C-termini structural information (PDB accession nos. 6VTL and 6VTK) to fit the intracellular domains into the open state model by homology modeling followed by minimization in Cartesian space with the RosettaMembrane energy function (Barth et al., 2007).

**Figure 2.**
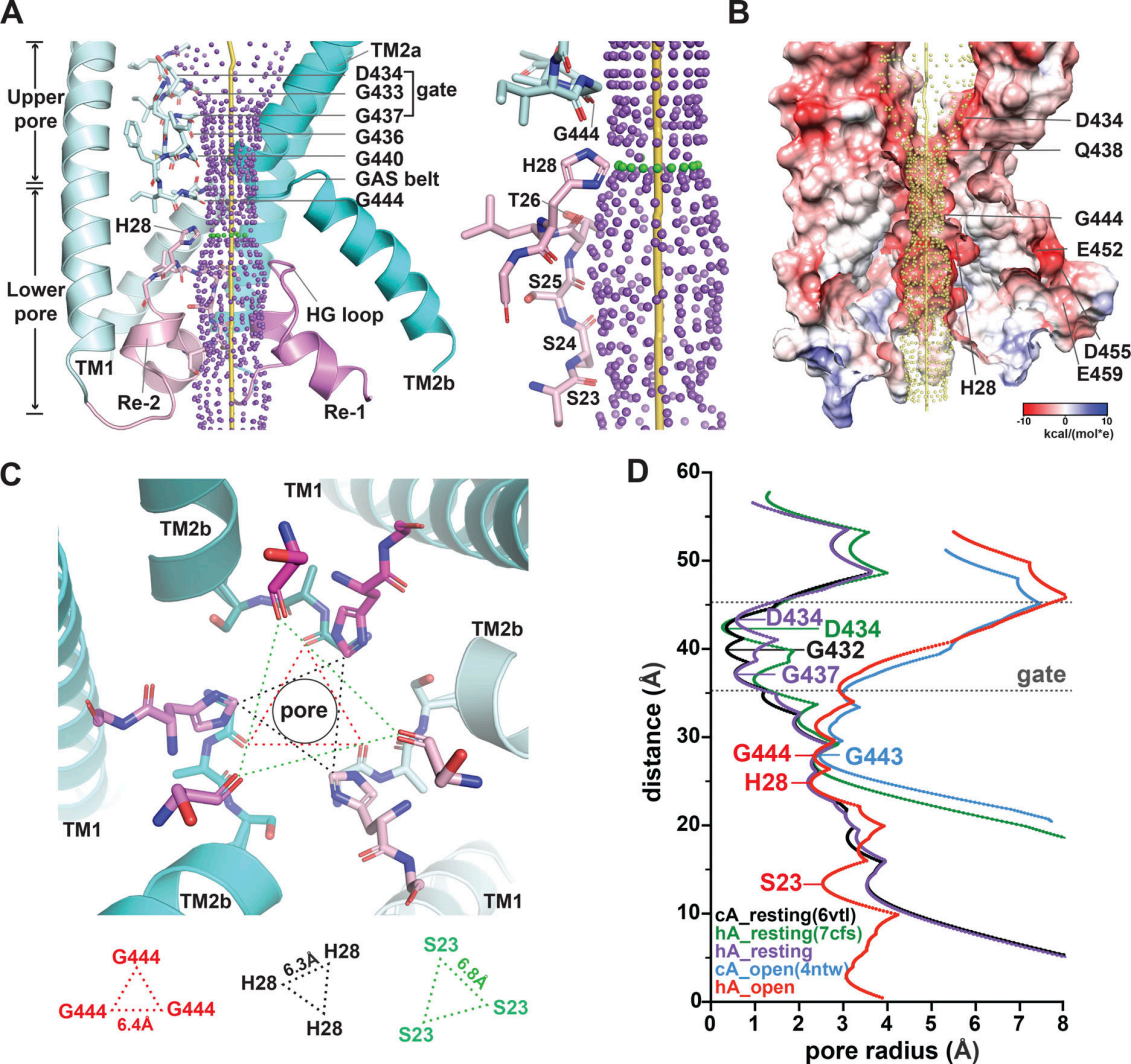
**Predicted structure of the open pore of hASIC1a and radius of the ion permeation pathway. (A)** Side view of the ion permeation pathway in the predicted open conformation of hASIC1a (left panel). Only two subunits are shown for clarity. The ion pathway is demarcated by purple dots. The TM domains are colored teal, and the reentrant loops are pink. An enlarged side view with residues lining the permeation pathway shows the following in sticks: G444, H28, T26, S25, S24, and S23 (right panel). **(B)** Side view illustration of the electrostatic potential distribution of the predicted hASIC1a open state model. Only two ASIC1 subunits and only the TM domains and the N-terminal reentrant loop are presented to visualize the interior surface of the central ion permeation pathway, which is demarcated by yellow dots. The coulombic electrostatic potential (ESP) was calculated by the built-in coulombic command in the UCSF Chimera system, with default coloring ranging from red for negative potential through white to blue for positive potential. **(C)** View from the cytosol of the ion pore in the predicted hASIC1a open conformation highlights the architecture and organization of the GAS belt of TM2 and the HG motif of the reentrant loop. **(D)** Profile of pore radius calculated by the HOLE2 program (Smart et al., 1996). hA_resting (7cfs), cryo-EM structure of hASIC1a at pH 8.0 (PDB accession no. 7CFS; Sun et al., 2020); hA_resting, predicted hASIC1a in the resting state; hA_open, predicted hASIC1a in the open conformation.

The closed and desensitized models of hASIC1a are very similar to the corresponding cASIC1 structures (PDB accession nos. 6VTL and 6VTK, respectively). The predicted pore structure of hASIC1a in the open state shows ∼20° rotation of TM1 and 30° rotation of TM2 compared with the closed state (Fig. 1, D–F), which is similar to the original open structure of cASIC1 in complex with MitTx (PDB accession no. 4NTW). The reentrant loop is also predicted to rotate ∼20° (i.e., it moves together with the TM helices such that the overall structure of the lower pore is kept quite similar in the closed and open states). Consequently, residues lining the ion permeation pathway are the same in closed and open conformations (Fig. 2 A and Fig. S2). Because opening of the pore rotates TM1 and TM2b, some side chain interactions of the TM domains with Re-1, Re-2, and the GAS belt are lost and replaced by new interactions formed in the open state. The impact of those predicted interactions on the stability of the open conformation and ion selectivity are examined in the ensuing sections.

The structure of the predicted hASIC1a open pore can be divided into upper and lower segments, with the GAS motif of TM2 being the limit between them (Fig. 2 A). The ion pathway of the upper pore is lined primarily by TM2a, and the lower section is made up almost entirely by the reentrant loop of the N-terminus. The three elements of the N-terminus—Re-1, HG loop, and Re-2—are enclosed by TM2b, the GAS belt, and TM1, respectively. In this location, the reentrant loop fills the large gap between TM1 and TM2b of the same subunit. H28 (HG motif) is the only side chain facing the aqueous pathway in the lower pore; other residues contribute with the backbone: G444-A445-S446 in the belt and S23 to L27 in Re-1, as shown in a side view of the open pore in Fig. 2 A. In this view, the yellow rod marks the center of the ion pathway, the purple dots delineate the surface of the pore, and residues lining the pore (teal for the GAS belt and pink for the reentrant loop) are shown in stick representation. A bottom view (Fig. 2 C) shows that the narrowest segment of the pore is defined by two rings made by the backbone of the GAS belt (pore radius ∼2.3 Å), and, right beneath it, the three imidazole side chains of H28 extend toward the center of the pore, delineating a second narrow constriction of ∼2.23 Å radius. At the end of the ion pathway, the carbonyl of S23 (Re-1) backbone slightly narrows the cytosolic entrance of the pore. The radius profiles of cASIC1 in resting and open conformations and of hASIC1a in predicted resting and open conformations are shown in Fig. 2 D.

The electrostatic potential distribution of the predicted hASIC1a pore in the open conformation shows the carboxylate of D434 (TM2a) in the upper section of the pore as the main contributor to the negative potential, whereas the backbone carbonyl oxygens of the reentrant loop are the main contributors in the lower section of the pore. Instead, the negative charges from residues E452, D455, and E549, all in TM2b, are situated far from the ion pathway (Fig. 2 B).

### A π–π interaction between F21 (Re-1) and Y456 (TM2b) tethers and stabilizes the Re-1 to TM2b

**Figure 3.**
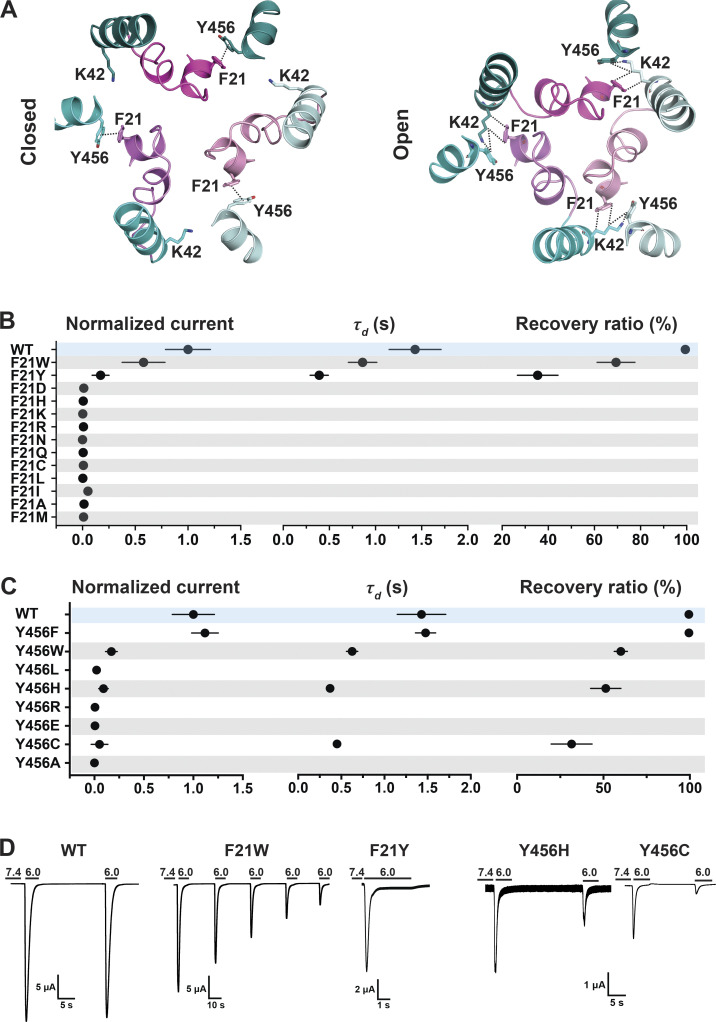
**A π–π interaction between F21 and Y456 stabilizes the resting conformation.** **(A)** Top view of the predicted hASIC1a in the closed and open conformations shows a π–π interaction between the conserved residues F21 (TM1) and Y456 (TM2b) in the resting state but not in the open conformation. TM2bs from the three ASIC1 subunits are presented in deep teal/cyan/pale cyan, while the three corresponding reentrant loops are displayed in light magenta/violet/light pink. **(B)** Summaries of normalized current, *τ*_d_, and recovery ratio of F21 substitutions. **(C)** From Y456 substitutions, only Y456W produced peak currents of a magnitude similar to WT (Dunnett’s P = 0.9), whereas all other mutants had P < 0.0001. **(D)** Representative current traces of WT hASIC1a, F21W, Y456H, and Y456C. Data are presented as mean ± SD of three independent experiments for at least five *Xenopus* oocytes (*n* = 5–10) for each construct tested. Oocytes were incubated for 30 s in preconditioning buffer (pH 7.4) before a second activation to measure the recovery ratio (expressed in percent), calculated as I_acti(n + 1)_/I_acti(n)_. Individual measurements and statistical analysis are shown in Data S2.

**Figure 4.**
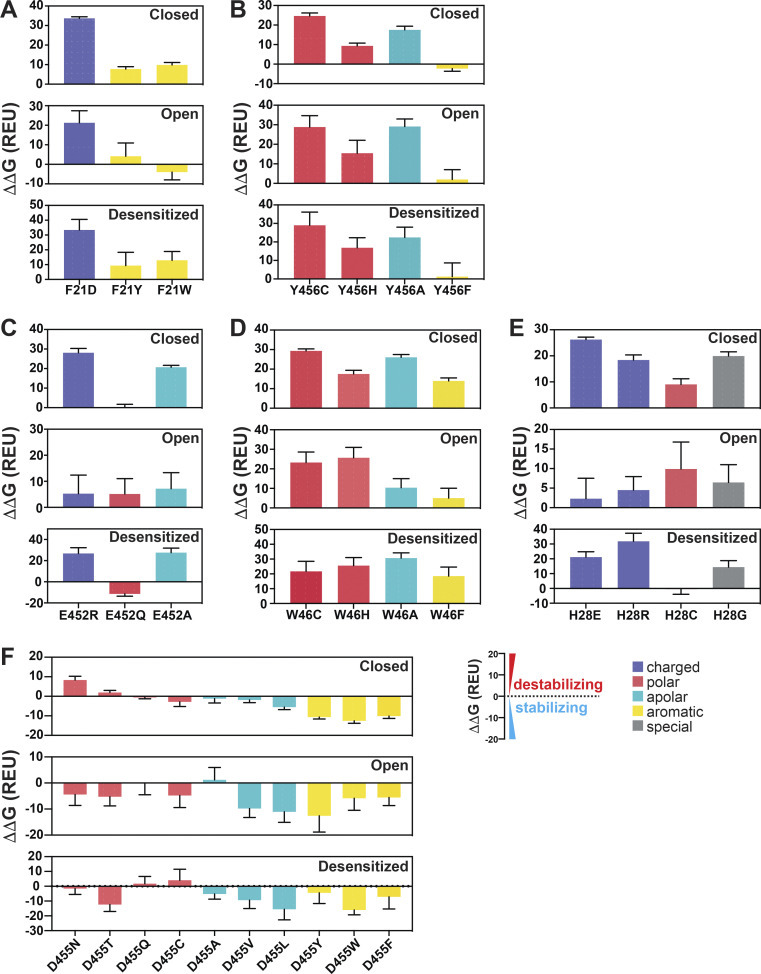
**Calculations of free energy changes (ΔΔG) of hASIC1a substitutions.** **(A–F)** Free energy changes (ΔΔG) calculations of hASIC1a substitutions of F21 (A), Y456 (B), E452 (C), W46 (D), H28 (E), and D455 (F) in the closed, open, and desensitized states compared with the WT hASIC1a in the corresponding conformations. A ΔΔG value >1 REU is considered to be destabilizing, while a ΔΔ*G* value less than −1 REU is considered to be stabilizing. All values are presented as mean ± SD. 10 runs of free energy calculations were conducted in replicates for each mutant and the WT hASIC1; 10 free energy values of the WT and mutants were sorted from smallest to largest before free energy values of the WT were subtracted from those of each mutant (10 ΔΔG values obtained for each mutant). Each error bar shows the SD of 10 ΔΔG values. For each amino acid substitution and the WT hASIC1a, the total free energy score was calculated from contributions of the individual score terms in the RosettaMembrane energy function to the stability of the closed, open, and desensitized state structures, respectively. Values of calculations are shown in Data S2. Breakdowns of Rosetta score terms applied in this study are listed in the following categories: LJ (Lennard-Jones), LK and Elec nonbonded: fa_atr, fa_rep, fa_sol, fa_intra_rep, fa_intra_sol_xover4, lk_ball_wtd, fa_elec; hydrogen bonding: hbond_sr_bb, hbond_lr_bb, hbond_bb_sc, hbond_sc; disulfide: dslf_fa13; backbone and sidechain geometry: omega, fa_dun, p_aa_pp, yhh_planarity, ref, sugar_bb, rama_prepro, cart_bonded. Detailed individual Rosetta score terms can be found at https://new.rosettacommons.org/docs/latest/rosetta_basics/scoring/score-types.

As indicated, the N-terminal reentrant loop is nested in a scaffold formed by the initial segment of TM1 and TM2b of the same subunit that holds in position the reentrant loop through side chain interactions. Upon opening, there is a reorganization of TM1 and TM2b leading to changes in some of the closed state–specific interactions. The amino acids F21 (Re-1) and Y456 (TM2b) form a π–π interaction in resting/desensitized states of cASIC1 (PDB accession no. 6VTL/6VTK; Yoder and Gouaux, 2020) and also in the predicted hASIC1a structures in the corresponding states (Fig. 3 A). Opening of the pore breaks the π–π interaction, placing the aromatic side chain of F21 facing C_β_H and C_δ_H from the K42 side chain of TM1 from the adjacent subunit (Fig. 3 A). To examine the functional significance of F21 and Y456 interactions, these residues were substituted by a series of other amino acids, as indicated in Fig. 3, B and C. Most substitutions abolish or reduce the magnitude of the peak current to ≤1 µA/cell; only two other aromatic residues produced currents W>>Y (Fig. 3 B and Data S2). Of note, because Y21 mutant exhibits fast desensitization (τ_d_ = 0.39 ± 0.10 s compared with WT 1.43 ± 0.28 s; P < 0.0001), it is likely that the value of the peak current was underestimated, owing to the slow solution exchange of the TEVC relative to channel kinetics. Residue Y456 also tolerates only aromatic substitutions: F>>W>H, with F being indistinguishable from WT and other enabling small currents with fast desensitization and partial recovery; many mutants do not recover full current after 1 min at pH 7.4 (Fig. 3 C and Data S2). In addition, calculated free energy changes (ΔΔG) with the Rosetta Flex ddG protocol (Barlow et al., 2018) and the Rosetta Membrane all-atom energy function (Barth et al., 2007) of F21 and Y456 mutants compared with WT hASIC1a in closed, open, and desensitized states show a destabilizing tendency for F21D and Y456C/H/A mutants in all three states (ΔΔG >1 REU) but not for Y456F (Fig. 4, A and B; and Data S2). The analysis suggests that residues F21 and Y456 are essential for proper channel function. The observation that only Y456F is able to keep full channel function implies that other factors not accounted for here (size of side chain, hydrophobicity, steric hindrance during the transition from closed to open) play important roles besides the stabilizing π–π interaction between residue F21^hASIC1a^/F22^cASIC1^ and Y456^hASIC1a^/Y455^cASIC1^ in closed and desensitized states.

### A state-dependent four-residue network tethers Re-2 to TM1 and TM2b

**Figure 5.**
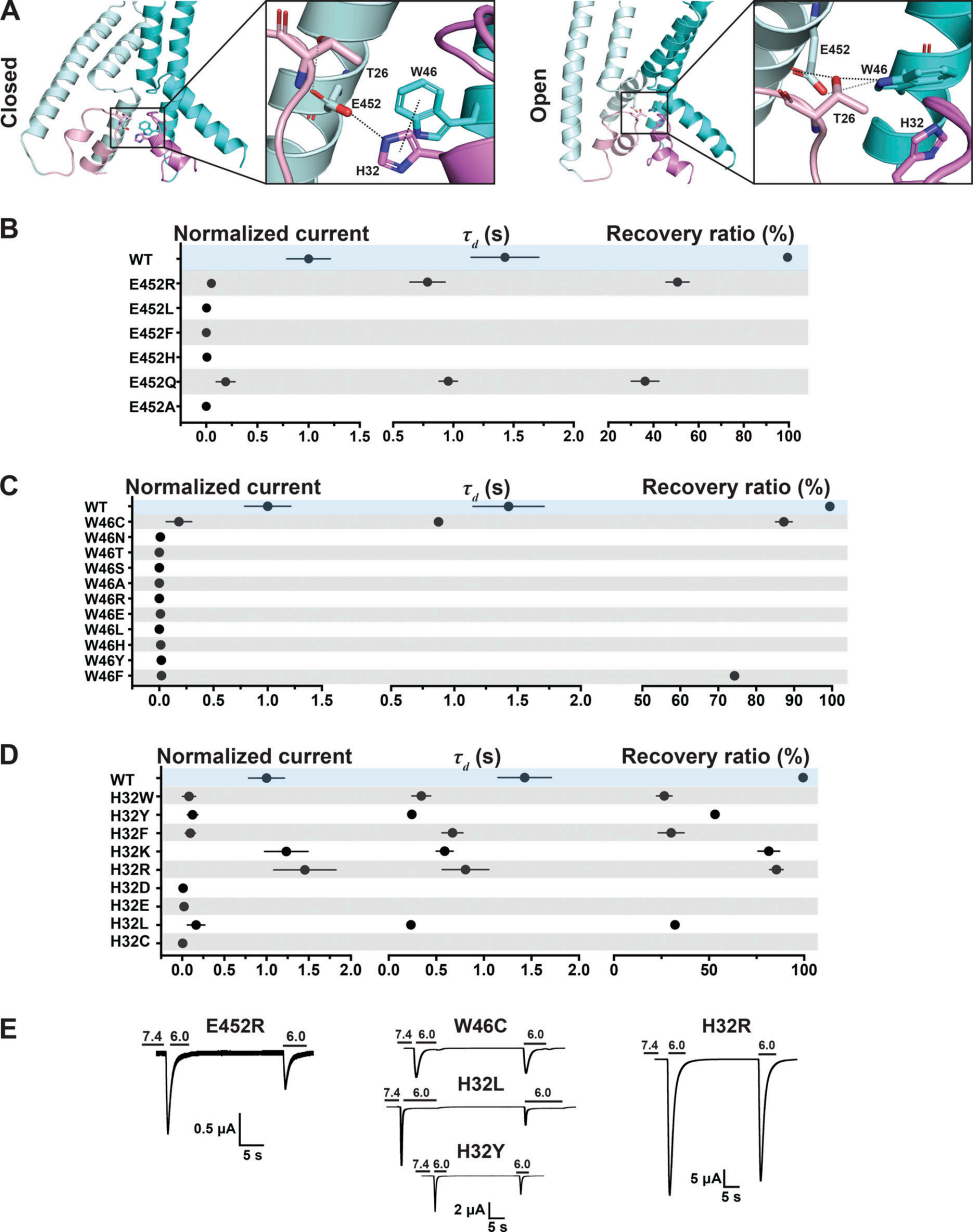
**A hydrogen bond between W46 and E452 stabilizes the predicted open conformation.** **(A)** Side view of the predicted hASIC1a in the resting (left panel) and open (right panel) conformations, highlighting interaction networks formed by residues E452 (TM2b), W46 (TM1), T26 (HG loop), and H32 (Re-2). Only two subunits are shown. TMs are in cyan/pale cyan, while corresponding reentrant loops are in violet/light pink. **(B)** Summaries of normalized current, *τ*_d_, and recovery ratio for E452. **(C and D)** W46 (C) and H32 (D) substitutions. **(E)** Representative traces of E452R, W46C, H32R, H32L, and H32Y. Data are presented as mean ± SD of three independent experiments for at least five *Xenopus* oocytes (*n* = 5–10) for each construct tested. All oocytes were incubated at least 30 s (30–40 s) in preconditioning buffer (pH 7.4) for channel recovery before the next activation. Recovery ratio (expressed in percent) was calculated as I_acti(n + 1)_/I_acti(n)_. Individual measurements and statistical analysis are shown in Data S2.

Transition from resting to open conformation predicts changes of a network of interactions between four highly conserved residues: H32 (Re-2), W46 (TM1), T26 (HG loop from the neighbor subunit), and E452 (TM2b from the neighbor subunit; Fig. 5 A). In the closed state, the carbonyl of the E452 side chain forms a hydrogen bond with the hydroxyl of T26, and the hydroxyl group of E452 forms another hydrogen bond with a nitrogen from the imidazole side chain of H32 of the adjacent subunit. H32 also interacts through a π–π interaction with W46 from the same subunit. Dashed lines in the enlarged view of the closed state in Fig. 5 A denote distances ≤4 Å. In the open state, E452 keeps the hydrogen bond with T26 but switches the interaction from H32 to the indole nitrogen of W46, and the π–π interaction is probably lost. Also in the open state, T26 gets closer to W64, forming a hydrogen bond with the indole nitrogen (enlarged view of the open pore in Fig. 5 A). Previous work indicated that W46 and E452 are essential residues to maintain channel function (Kasimova et al., 2020; Lynagh et al., 2020, 2017; Yang and Palmer, 2018). Here, we tested additional substitutions of residue E452: R/L/F/H/Q/A. All mutants were nonfunctional or had small currents (Fig. 5 B and Data S2). The only substitution that retained some function was E452Q, though currents were significantly smaller than WT (0.21 ± 0.1; P < 0.0001 compared with WT), desensitized fast (τ_d_ 0.96 ± 0.076 s; P = 0.01), and partially recovered from desensitization (36.26 ± 6.13%; P < 0.0001). The multiple and different interactions E452 makes in closed and open states could explain why this residue is so essential to stabilizing the closed and open conformations. Fig. S3, A and B, shows that Q452, the only substitution with some degree of function, still interacts with T26 and H32 in the closed state but cannot interact with W64 in the open state, providing an explanation for why only Q452 can work as a partial substitute.

Electrophysiological studies also confirmed the importance of the indole group of W46 for channel function (Fig. 5 C and Data S2). Most of the substitutions examined (W46/N/T/S/A/R/E/L/H/Y/F) produce very small currents (≤0.5 µA/cell); only W46C exhibits currents of average magnitude 4.55 ± 2.28 µA/cell (P < 0.0001), though channels desensitize fast (τ_d_ 0.87 ± 0.012 s; P < 0.0001) and recover partially after the first stimulus (87.3 ± 2.19%; P < 0.0001). Calculations of free energy changes of E452 mutants in three states compared with those of WT hASIC1a produce values of ΔΔG >1, consistent with destabilization of all three states for E452R/A but not for E452Q (Fig. 4 C and Data S2). For residue W46 mutants, W46C/H/A/F were all destabilizing (Fig. 4 D and Data S2).

Similarly, most H32 mutants exhibit small currents with rapid desensitization and incomplete recovery after the first stimulus. Only H32K and H32R have currents of a magnitude similar to those of WT channels (Fig. 5, D and E; and Data S2), though desensitization of H32K is faster (τ_d_ = 0.59 ± 0.089 s; P < 0.0001), suggesting that this position favors amino acids with side chains carrying a positive charge, possibly because a Lys and Arg at this position could maintain the interaction with E452 via a salt bridge and with W64 via a π–cation interaction stabilizing the resting conformation.

### Interactions of the HG loop with the GAS belt and TM2b

**Figure 6.**
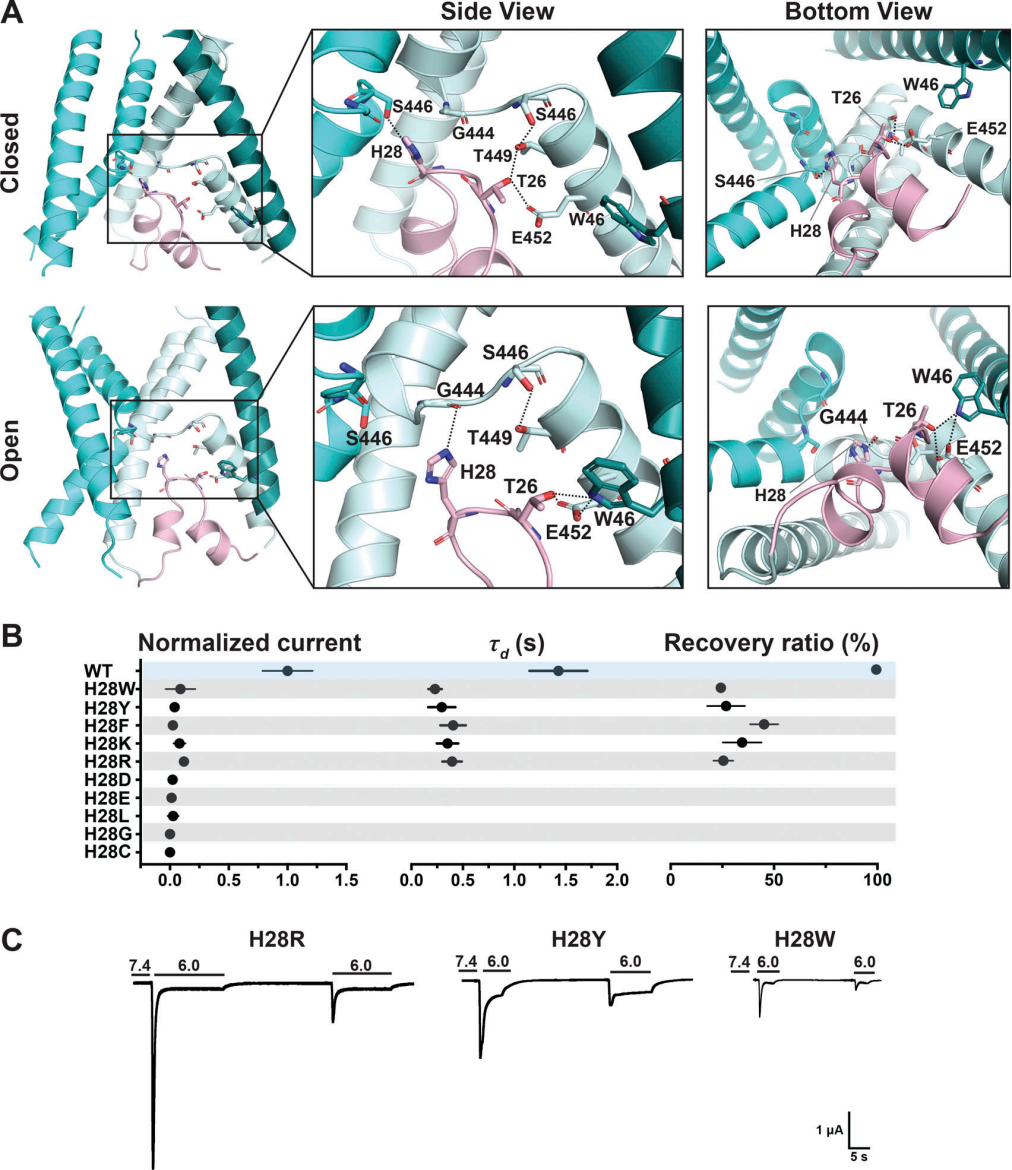
**H28 forms state-dependent stabilizing interactions with the GAS belt and T26 with TM1 and TM2b.** **(A)** Side and bottom views of hASIC1a in the predicted resting (upper panel) and open (lower panel) conformations, highlighting interaction networks formed by residues H28, T26 (HG loop), and the lower pore. Three TM1 helices (deep teal/cyan/pale cyan), two TM2 helices (cyan/pale cyan), and one reentrant loop (light pink) are shown. **(B)** Summary of normalized current, *τ*_d_, and recovery ratio for H28 substitutions. All mutants produce significantly smaller currents than WT (P < 0.00001), have faster desensitization (P < 0.0001), and partial recovery (P < 0.0001). **(C)** Representative traces of H28R, H28Y, and H28W. Data are presented as mean ± SD of three independent experiments for at least five *Xenopus* oocytes (*n* = 5–10) for each construct tested. All oocytes were incubated at least 30 s (30–40 s) in preconditioning buffer (pH 7.4) for channel recovery before the next activation. Recovery ratio (expressed in percent) was calculated as I_acti(n + 1)_/I_acti(n)_. Individual measurements and statistical analysis are shown in Data S2.

The HG motif in the loop connecting Re-1 and Re-2 is highly conserved in ASICs and ENaC channels. In the predicted models of the hASIC1a lower pore in closed and open conformations, the side chain of H28 faces the ion pathway in a narrow segment of the pore, suggesting its function may relate mainly to ion permeation and selectivity. Further analysis indicates that H28 interacts with residues in the GAS belt (G444-A445-S446). In the closed state, it forms a hydrogen bond with the side chain of S446 of a neighboring subunit (-NH with -OH), whereas in the predicted open conformation, H28 interacts with the main chain carbonyl of G444 in the same subunit and no longer with S446. Another residue that tethers the HG loop to TM2b is T26, which forms intrasubunit hydrogen bonds with T449 and E452 (both in TM2b) in the closed state but changes interactions in the open conformation to W64 (neighbor subunit) while keeping the hydrogen bond with E452 (Fig. 6 A).

The electrophysiological results show that all tested substitutions of H28W/Y/F/K/R/D/E/L/G/C abolish or markedly decrease currents (Fig. 6, B and C; and Data S2). The mutation with the largest current is H28R that expresses only 11 ± 2.03% of the WT currents and desensitizes fast (τ_d_ = 0.38 ± 0.099 s; P < 0.0001) with little recovery (25.5 ± 4.75%; P < 0.0001). The finding of Arg able to generate currents despite exposing a large and positively charged side chain to the center of a narrow section of the pore lumen was unexpected; the result is revisited in a following section when we discuss effects of these substitutions on channel selectivity. Calculated free energy changes (ΔΔG) of H28 mutants compared with the WT channel in resting, open, and desensitized states predict destabilization of the closed state in all three states for H28E/R/C/G (Fig. 4 E and Data S2). All the results from H28 support the notion that this highly conserved residue is essential and that its interactions with the GAS belt mutually stabilize the two loop structures in the central pore.

### Lower pore residues contribute directly and indirectly to set ion selectivity

The determinants of ion selectivity in ASICs are not yet known and remain a topic of ongoing investigation. Current candidates are the GAS belt and two negative charges in TM2b (E452/D455; Lynagh et al., 2017; Yang and Palmer, 2018), and the recently solved structure of the reentrant loop has added H28 as a third candidate (Yoder and Gouaux, 2020).

**Figure 7.**
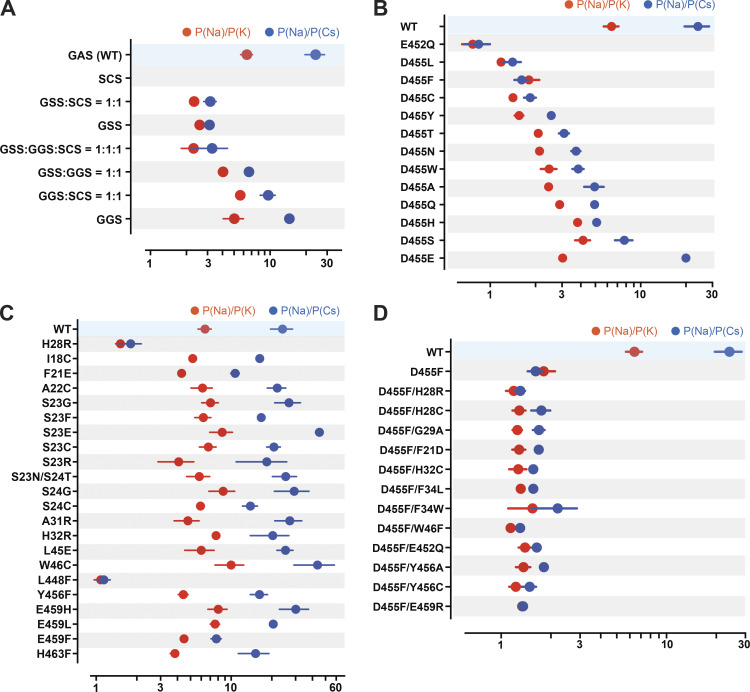
**Ion selectivity of hASIC1a lower pore mutants.** **(A)** Summaries of changes in the ion selectivity sequence, P(Na):P(K):P(Cs), of GAS belt mutants. All combinations shown have significant decreased selectivity compared with WT by one-way ANOVA post hoc Dunnett’s test (P < 0.0001), except the P(Na):P(K) ratio of GGS:SCS = 1:1 and GGS P = 0.33 and 0.026, respectively. **(B)** E452 and D455 mutants all display significant lower selectivity than WT (P < 0.0001). Selectivity of D455E taken from Lynagh et al. (2017). **(C)** H28R, W46C, L448F, and other lower pore mutants; among them, only H28R and L448F have significant lower selectivity than WT (P < 0.0001). **(D)** D455F rescues currents from nonfunctional mutants, but the ion selectivity of all the double mutants is low compared with WT (P < 0.0001). For measurement of reversal potential of Na^+^, K^+^, or Cs^+^ for WT and mutants, the external solution was changed from the preconditioning buffer containing 100 mM of either NaCl/KCl/CsCl, 5 mM HEPES, 5 mM Mes, and 2 mM CaCl_2_, pH 7.4, to the activating buffer containing either 100 mM NaCl/KCl/CsCl, 5 mM HEPES, and 5 mM Mes, pH 6.0. Reversal potential of Na^+^, K^+^, or Cs^+^ was calculated from differences in the reversal potential of currents measured from a −80-mV to +60-mV voltage ramp by TEVC with the indicated cations in the external solutions. Data are presented as mean ± SD of two independent experiments for at least five activations from 3 to 10 *Xenopus* oocytes (*n* = 5–10) for each ion condition and for each construct tested. For hASIC1a mutants including H28R, E452Q, D455S/H/T/N, and E459F that exhibit small peak current with reduced recovery from desensitization, reversal potential of Na^+^, K^+^, or Cs^+^ was calculated only with measured activated peak current ≥3 µA/cell. Individual values and statistical analysis are shown in Data S2.

Here, we looked for residues likely involved in ion selectivity using electrophysiological studies and the predicted open structure of hASIC1a to bridge function to structure. First, we examined the GAS belt. Substitution of the first two residues in hASIC1a by its counterparts in ENaC (a close relative of ASICs but with higher Na selectivity of P_Na_/P_K_ >100/1; Yang and Palmer, 2018): Gly-Ser-Ser (GSS) from αENaC, Gly-Gly-Ser (GGS) from β-ENaC, and Ser-Cys-Ser (SCS) from γ-ENaC. Owing to very small currents of SCS^hASIC1a^, the ion selectivity of this mutant was not measured. Fig. 7 A and Data S2 show that GSS^hASIC1a^ decreased the ion selectivity of Na^+^ over K^+^ and Cs^+^, and GGS^hASIC1a^ was similar to that of WT; nevertheless, these mutants expressed decreased currents, accelerated desensitization, and did not recover fully from desensitization (Fig. S4 A and Data S2). Coexpression of subunits in the following ratios (GSS^hASIC1a^:GGS^hASIC1a^ = 1:1, GSS^hASIC1a^: SCS^hASIC1a^ = 1:1, GGS^hASIC1a^: SCS^hASIC1a^ = 1:1, or GGS^hASIC1a^:GSS^hASIC1a^:SCS^hASIC1a^ = 1:1:1) also display various degrees of decreased ion selectivity. Calculations of free energy changes of these mutants compared with the WT hASIC1a in resting, open, and desensitized states showed destabilization of the three states (ΔΔG >1 REU; Fig. S4 B and Data S2), consistent with the observed changes in kinetics. The three residues of the belt accommodate only small side chains, owing to steric hindrance in this section of the pore. Additionally, the side chain of S446 forms a stabilizing bond with H28 (2.86 Å) in the closed state and with T449 in the closed (3.2 Å) and open (2.9 Å) states, as indicated previously in Fig. 6 A. We conclude that G444 and S446 are essential to maintain the function of hASIC1a, whereas A445 can be partly substituted by G and S but with impairment of channel function. These results together with previous publications support that the GAS belt contributes to the ion selectivity of hASIC1a and also plays a crucial role in keeping the pore stable through interactions with residues of the lower pore (Carattino and Della Vecchia, 2012; Kellenberger et al., 2001, 1999a, 1999b; Kellenberger and Schild, 2002; Lynagh et al., 2017; Yang and Palmer, 2018).

Two highly conserved negatively charged residues, E452 and D455, have been reported to constitute the selectivity filter (Lynagh et al., 2020, 2017; Yang and Palmer, 2018). In our studies, E452 is highly sensitive to mutations, which markedly decrease or abolish currents. We were able to measure ion selectivity confidently only in the mutant E452Q by selecting cells with currents ≥4 µA/cell (mean, 4.54 ± 2.17 µA; Fig. 5 B and Data S2). E452Q completely lost selectivity for Na^+^, K^+^, and Cs^+^ (Fig. 7 B and Data S2).

**Figure 8.**
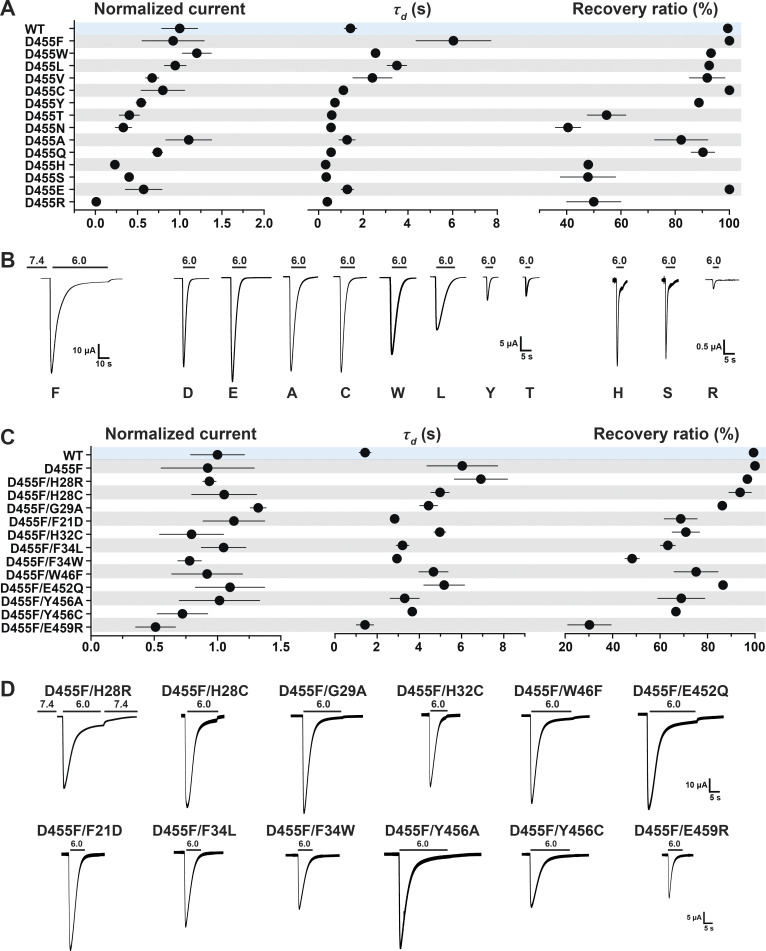
**A Phe at position D455 stabilizes the lower pore and rescues destabilizing nonfunctional lower pore mutants. (A)** Summary of normalized current, *τ*_d_, and recovery ratio of various D455 substitutions. **(B)** Representative examples of current traces of D455 substitutions. **(C)** Summary of normalized current, *τ*_d_, and recovery ratio of double mutants (D455F and an additional substitution in the lower pore). **(D)** Representative examples of current traces of the double mutants. Data are presented as mean ± SD of three independent experiments for at least five *Xenopus* oocytes (*n* = 5–10) for each construct tested. All oocytes were incubated at least 30 s (30–40 s) in preconditioning buffer (pH 7.4) for channel recovery before the next activation. Recovery ratio (expressed in percent) was calculated as I_acti(n + 1)_/I_acti(n)_. Individual measurements are shown in Data S2.

In contrast, residue D455 tolerates many substitutions, some of which yield currents of magnitude comparable to those of WT channels, as shown in Fig. 8, A and B (and the Data S2), but all are nonselective with the exception of D455E (Fig. 7 B and Data S2). The mutants D455F/W/L/V also exhibit slower desensitization than WT. Among them, D455F has the slowest desensitization rate (*τ*_d_ = 6.04 ± 1.68 s; P < 0.0001), followed by D455L (*τ*_d_ = 3.51 ± 0.43 s; P < 0.0001); in addition, D455F exhibits a component of sustained current (I_sust_/I_max_ = 5%; Fig. 8 B). Analysis of unitary currents from patches expressing D455F reveals long opening events (200–2,000 ms versus WT mean duration of 30 ms as previously determined; Chen et al., 2021; Fig. S5, B and D). Notably, when D455F was added to a series of other mutants with levels of current too small to be reliably evaluated, the double mutants markedly increased the magnitude of currents, slowed the rate of desensitization, and increased recovery from desensitization (Fig. 8, C and D, and Data S2). All the double mutants were also nonselective (Fig. 7 D and Data S2). Furthermore, double mutants made of D455F and a second mutation, either H28R/C or H32C, exhibit higher apparent proton affinity for activation: D455F pH_50a_ 6.76, D455F/H28R pH_50a_ 7.00, D455F/H28C pH_50a_ 7.03, and D455F/H32C pH_50a_ 6.99 versus WT pH_50a_ 6.68; all values were statistically significantly higher than WT (P < 0.0001; Fig. S6 and Data S2). Analysis of unitary currents from patches expressing D455F/H28R also revealed long opening events (2–10 s versus WT mean duration of 30 ms as previously determined; Chen et al., 2021). Unitary currents retained the same magnitude of WT channels (Fig. S5, C and D, and Data S2). Together, these findings indicate that D455F stabilizes the open conformation, and this effect rescues other nonfunctional lower pore mutants (Fig. 8, C and D). Furthermore, the shift of pH_50a_ to more alkaline values suggests that the double mutations increase the efficacy of protons (i.e., facilitate proton-mediated openings besides stabilizing the open conformation).

Calculations of free energy changes of residues with hydrophobic side chains, D455V/L/W/F, compared with WT hASIC1a in resting, open, and desensitized states favor stabilization (ΔΔG less than −1 REU; Fig. 4 F and Data S2), consistent with the electrophysiological data: large currents, slow desensitization, and >90% recovery (Fig. 8, A and B, and Data S2).

Structural evidence of cASIC1 has made apparent that the side chains of E452 and D455 are not in contact with the ion permeation pathway in closed and desensitized states, and our model of hASIC1a predicts they also may not be exposed in the open conformation. We indicated that E452 is an essential component of an interaction network involving the reentrant loop, TM2b and TM1 (Figs. 5 A and 6 A), which supports the GAS belt. Thus, in the instance of E452, the mechanism underlying reduced ion selectivity is likely indirect and involves stabilization of the GAS and HG motif in the center of the pore. Different from E452, the side chain of D455 faces the lipid bilayer and TM1 of the neighboring subunit. In the closed state, it interacts with K42 (TM1), and in the open state, it may interact with C49 (TM1; Fig. S7). Replacement by Phe may strengthen the interaction with the lipid bilayer, stabilizing the open conformation; however, an explanation for the allosteric process that leads to loss of selectivity of D455F remains elusive.

### De novo models of nonselective hASIC1a mutants in the open conformation

**Figure 9.**
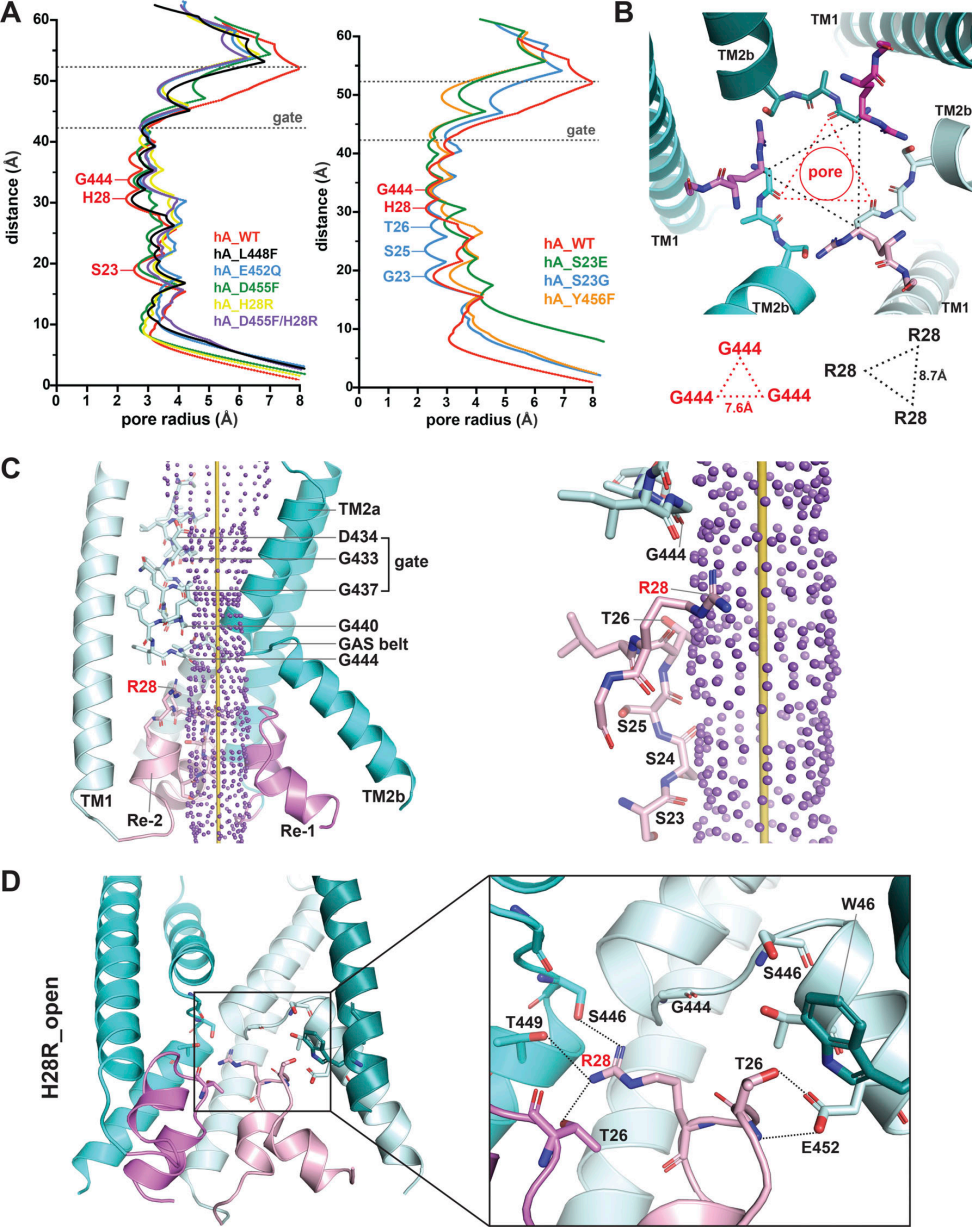
**Predicted structures of hASIC1a mutants in the open conformation exhibit enlarged pore radii and altered interaction networks in the lower pore.** **(A)** Right: Pore radius profile of WT hASIC1a compared with nonselective mutants. Left: Pore radius profile of WT hASIC1a compared with selective mutants using HOLE2 software. **(B)** Bottom view of R28^hASIC1a^ ion pore in the predicted open conformation, highlighting the architecture and sizes of the rings defined by the GAS belt and R28 in the reentrant loop. **(C)** Side view of the ion permeation pathway of predicted R28^hASIC1a^ in the open conformation (left panel) and an enlarged view of the ion permeation pathway with residues in stick representation lining the ion pathway: G444, R28, T26, S25, S24, and S23 (right panel). **(D)** Side view of predicted R28^hASIC1a^ pore in the open conformation (left panel) highlights altered interaction networks formed by residues R28, T26, and the lower pore (right panel). Three TM1 helices (deep teal/cyan/pale cyan), two TM2 helices (cyan/pale cyan), and reentrant loops (violet/light pink) are shown for simplicity.

Next, we asked whether there is a distinct structural change in nonselective mutant channels that is not present in other lower pore mutants that remain ion selective. To that end, we modeled de novo L448F, E452Q, D455F (all three in TM2b), H28R, and the double mutant H28R/D455F in the open conformation. These mutations were selected because all induce loss of ion selectivity, and, with the exception of H28R, they do not face the pore lumen. The radius profiles of the predicted open pore of all these mutant channels show increased radius at the level of the GAS belt and the HG motif compared with the WT (Fig. 9 A).

Mutations of H28 have been shown to alter ion selectivity (Sheikh et al., 2021). Among the numerous substitutions tested here (Fig. 6 B), H28R exhibits sufficiently large currents in the first activation (4 µA compared with ≤1 µA) to enable reliable measurements of reversal potential showing that it is not selective (Fig. 7 C). In the predicted R28^hASIC1a^ open conformation, both the GAS belt (from a pore radius of 2.3 Å in the WT to 3.01 Å in H28R mutant) and R28 constriction (from a pore radius of 2.23 Å in the WT to 3.59 Å in H28R mutant) are expanded. The side chains of the three Arg are located right beneath the GAS belt, but they do not form a constriction of the ion permeation pathway: the side chain of R28 does not point directly to the center of the pore, but it is parallel to the perimeter of the pore (Fig. 9, B and C). The network of interactions of H28 differs from that of R28. While H28 interacts with G444 (GAS belt of same subunit), R28 interacts with other residues: S446 (GAS belt), T449 (TM2b), and T26 (HG loop), all from the neighbor subunit (Fig. 9 D). Additionally, the absence of the stabilizing hydrogen bond between W46 and E452 likely contributes to the low stability of R28^hASIC1a^ channels in the open conformation. As a control of de novo modeling of nonselective mutants, we also conducted additional models of functional and selective mutants: S23E/G and Y456F. None of them changed significantly the pore radius at the GAS belt (Fig. 9 A), indicating that not all amino acid substitutions in the lower pore predict hASIC1a structures with enlarged pore size.

## Discussion

This work explores the contribution of conserved residues in the lower pore of hASIC1a in maintaining the stability of the open conformation and ion selectivity. To understand the molecular mechanisms underpinning these properties, electrophysiological results were interpreted in light of predicted structures of hASIC1a in resting, open, and desensitized conformations that were modeled using cASIC1 with the N-terminus reentrant loop in the lower pore. Although we recognize the limitations of models, it is accepted that predicted models and experimentally resolved structures are complementary, and both have strengths and limitations. For a protein structure to be a gold standard, it has to be of high resolution and cannot be distorted by artifacts introduced by cryo-EM or crystallography procedures. On the other hand, predictions are becoming very accurate. We compared our model of hASIC1a in closed state obtained by Rosetta algorithms with the published one on the AlphaFold Protein Structure website (https://alphafold.ebi.ac.uk). The two models are almost identical, even though different algorithms were used, which lends confidence to our model. In this study, the electrophysiological results were largely in agreement with the predicted structures such that the models were instrumental in proposing possible mechanisms of gating and ion permeation. However, in a few instances, the models failed to explain functional effects of substitutions (e.g., F34 [Re-2]; Fig. 1 C) allowed only by Y/W (Data S2, F34 mutants), but F34 does not interact with other residues in the predicted structures; thus, the requirement of an aromatic side chain at this position was not identified, because the position of the side chain is incorrectly predicted and/or because of other unaccounted for factors. Also, the molecular mechanism underlying enlargement of the GAS belt and loss of ion selectivity mediated by D455F was not apparent, as previously indicated.

### Predicted pore structure

The structure of the TM helices in the modeled hASIC1a lower pore in open conformation is similar to that of cASIC1 in the open state (PDB accession no. 4NTW), while the reentrant loop has a structure that resembles but is not identical to that of cASIC1 in the closed/desensitized states (PDB accession nos. 6VTL/6VTK). The TM domains and reentrant loop are held together by networks of side chain interactions between Re-1/TM2b, Re-2/TM1/TM2b, and HG loop/GAS belt/TM1/TM2b. These interactions differ in the open and closed states as a consequence of the iris-like rotation of TM1 and TM2b upon opening (Fig. 1, D–F). Though the reentrant loop also rotates together with the TM helices, the rotation does not completely follow the motion of the helices, inducing rupture of contacts and formation of new ones within the scaffold of TM1 and TM2b. Because most of the ion pathway in the lower pore is lined by residues from the reentrant loop, the predictions suggest that the pore of hASIC1a is not a fixed structure, but it reshapes when the channel opens.

The main features of the predicted structure of the lower pore in the open conformation are shown in Fig. 2. Midway through the thickness of the membrane, there is a constriction in the ion pathway formed by the GAS belt (2.3 Å), and, right underneath it, the side chain of H28 in the loop connecting Re-1 and Re-2 forms another constriction (2.23 Å) of closely the same dimension as the GAS belt; these values are too similar to state with confidence which is the narrowest segment of the pore. Down the constrictions follows the backbone of residues from T26 and Re-1 (T26 to S23). Together they line the ion pathway of the lower pore in the predicted open conformation.

### Ion selectivity

A recurrent finding from the functional analysis of mutations in various regions of the lower pore and from modeling of those mutant channels is that the GAS belt and the HG motif are not rigid structures; their shapes/dimensions are altered by local and distal amino acid substitutions. It likely happens through a web of molecular interactions that includes direct links between residues of the GAS belt with the HG loop and more indirectly with residues in TM1 or TM2b. Accordingly, the ion selectivity of hASIC1a is not determined exclusively by the amino acid sequence of the “selectivity filter,” but it is modified by the context (i.e., surrounding sequences). This idea is illustrated in the experiment where we replaced the sequence GAS of hASIC1a by the sequences of the three corresponding motifs of the three subunits of ENaC, which exhibit high selectivity of Na^+^ over K^+^ ≥100. None of the tested combinations increased the hASIC1a channel preference for Na^+^; on the contrary, the selectivity of Na^+^:K^+^ decreased or remained similar to that of hASIC1a. This experiment is the counterpart of the one performed previously by Yang and Palmer (2018), where the authors introduced the GAS sequence of ASIC1 in the three subunits of ENaC. The result was a higher selectivity ratio of Li^+^:Na^+^ and smaller conductance of Na^+^ compared with WT. Thus, in both instances, the sequence of the three residues that form the belt of TM2 does not determine selectivity by itself, likely because these amino acids contribute to the ion pathway with the backbone rather than the side chains. The most parsimonious explanation is that the belt is not a rigid structure, but its diameter is modified by residues that interact directly and indirectly with this structure, among them the reentrant loop. The notion that the GAS belt is distensible is also supported by a greater pore radius in crystals of the cASIC1–MitTx complex in the presence of Cs^+^ (4.0 Å) than in the presence of Na^+^ (3.5 Å; Baconguis et al., 2014).

The question whether the GAS belt or HG motif constitutes the selectivity filter cannot be definitively answered by our electrophysiological results, because amino acid substitutions in either of these structures always lead to changes in selectivity and kinetics. This arises because the structures of these motifs are tightly interconnected in closed and open states. A subtler perturbation that preserves the side chains but changes properties of the backbone was used by the Pless study group: replacing the amide by an ester bond between the conserved glycine and alanine of the GAS increased Na^+^ over K^+^ selectivity of mASIC1a (Lynagh et al., 2017). This is consistent with the backbone of the GAS belt having an important role in determining ion selectivity. In addition, mutations that increase the size of the pore at the level of the HG motif still preserve ion selectivity in our predicted structures (Fig. 9), suggesting that the GAS belt may be sufficient to determine hASIC1a selectivity.

The structure of the cASIC1–MitTx complex in the open conformation and our predicted hASIC1a in the open conformation show that the ion pathway is lined almost entirely by the backbone of residues in TM2 and the reentrant loop, only the side chains of D434 and Q438 in the upper pore and of H28 in the lower pore point to the lumen. A charge density map of the surface of the predicted open pore (Fig. 2 B) does not have sites with strong electronegativity; rather, it is lined by a diffusely negative electrostatic potential given by the main chain carbonyls of residues lining the pore. Accordingly, the selectivity series: Na^+^ > K^+^ > Cs^+^ seems to originate primarily from the size of the filter with lesser contribution from electrostatic interactions between the permeant ions and charged groups. The hypothesis that the two negatively charged residues in TM2b, E452 and D455, participate in determining preferential Na^+^ selectivity (Lynagh et al., 2017) is unlikely in view of the most recent structures of cASIC that include the N-terminal reentrant loop (Yoder and Gouaux, 2020). These structures show that E452 and D455 are not in contact with the permeation pathway in the closed/desensitized or the predicted open conformation in this study. Even though our prediction of hASIC1a structure in open conformation might not be exactly the same as the yet to be experimentally determined structure, it is highly unlikely that the reentrant loop, which lines all the lower pore, falls out from the open pore to expose the side chains of E452 and D455 to permeant ions.

Taken together, the results presented here are consistent with the close-fit mechanism governing selectivity wherein the backbone of the GAS substitutes the hydration shell of a permeant ion to enable crossing the pore constriction. Though sieving may be the principle of selectivity, permeation of ions is affected by other factors. It has been shown that ENaC has a site—not yet identified—in the upper section of the pore that binds Na^+^ with two to three times higher affinity than that of K^+^, and it contributes to preferential Na^+^ conductance (Palmer and Frindt, 1988). hASIC1a has a negatively charged side chain exposed to the ion pathway (D434) that contributes to increasing the local concentration of Na^+^ in the outer segment of the pore and increases unitary conductance (*K*_m_ 25 mM; Yang and Palmer, 2014).

### Stability of the open conformation

Most substitutions of conserved residues in the lower pore examined here destabilize the open conformation, indicated by fast shutting of the pore (small τ_d_ values). They also negatively impact the closed/desensitized states denoted by slow or incomplete recovery from desensitization. In many cases, this effect was severe, preventing accurate measurement of pH_50a_ and of reversal potential (both require multiple sequential activations with low pH). The electrophysiological results were supported in most instances by predictions of free energy changes that yielded ΔΔG values >1 REU. Together these findings underscore the importance of interactions between elements of the lower pore: TM helices and the reentrant loop to keep the stability of the pore in both open and closed/desensitized states. The exception was residue D455 that, when replaced by hydrophobic residues, slowed the rate of desensitization and lengthened the duration of opening events. D455F produced the highest degree of stabilization of the open pore, complete recovery from desensitization, and a slightly higher apparent proton affinity for activation, suggesting that this mutation has a dual effect: it stabilizes the open conformation and increases the efficacy of proton gating. The side chain of D455 faces TM1 and the hydrophobic environment created by the lipid bilayer (Fig. S7 B), which is expected to destabilize WT channels, whereas substitution by a hydrophobic side chain is stabilizing, consistent with the kinetic properties exhibited by F455^hASIC1a^. The loss of ion selectivity of F455^hASIC1^ was unexpected because this residue is not in the ion pathway and does not interact with residues connected to the GAS belt or reentrant loop. Nevertheless, the predicted model of F455^hASIC1^ in open conformation shows widening of both the GAS belt and H28 constriction as other mutant channels with loss of ion selectivity. We can only speculate that, upon opening, F455 may alter movement of TM2b, straining the GAS belt.

A consequence of the complex interactions of residues in the lower pore with those in the GAS and HG motifs is that the ion selectivity and stability of the state conformations are interdependent. To attain high Na^+^ selectivity and also high stability of the open state, as in the case of ENaC, a superior choice may be to use combinations of different subunits. Such heteromeric channels could ensure favorable interactions with one neighbor subunit to achieve high Na^+^ selectivity and counteract any penalty in stability by having a different interaction with other neighbor subunits. This could be a potent selection force in favor of the heteromeric structure of ENaC, in which the subunits exhibit only ∼35% amino acid identity (overall amino acid identity α:β = 34.6%, α:γ = 36%, and β:γ = 36.9%). In contrast, the identity of the most abundant ASIC subunits, ASIC1a and ASIC2, is 72%.

## Supplementary Material

Data S1 is a source data file of ΔΔG.

Data S2 is a data file of all electrophysiological measurements shown in the figures.

Data S3 lists Rosetta commands and scripts used to obtain the predicted open, closed, and desensitized structures of hASIC1a.
