# Supplementary material for: A flexible GAS belt responds to pore mutations changing the ion selectivity of proton-gated channels
Source: J Gen Physiol. 2021 Nov 12;154(1):e202112978. doi: 10.1085/jgp.202112978 (PMC8594623; doi:10.1085/jgp.202112978)
Supplement: Data S3 — lists Rosetta commands and scripts used to obtain the predicted open, closed, and desensitized structures of hASIC1a. [file JGP_202112978_DataS3.docx]

**1. RosettaCM and relax**

**1.1 RosettaCM**

-in:file:fasta target.fasta

-parser:protocol rosetta_cm.xml

-nstruct 100

-relax:minimize_bond_angles

-relax:minimize_bond_lengths

-relax:jump_move true

-default_max_cycles 200

-relax:min_type lbfgs_armijo_nonmonotone

-relax:jump_move true

-score:weights stage3_rlx_membrane.wts

-use_bicubic_interpolation

-hybridize:stage1_probability 1.0

-chemical:exclude_patches LowerDNA UpperDNA Cterm_amidation SpecialRotamer VirtualBB ShoveBB VirtualDNAPhosphate VirtualNTerm CTermConnect sc_orbitals pro_hydroxylated_case1 pro_hydroxylated_case2 ser_phosphorylated thr_phosphorylated tyr_phosphorylated tyr_sulfated lys_dimethylated lys_monomethylated lys_trimethylated lys_acetylated glu_carboxylated cys_acetylated tyr_diiodinated N_acetylated C_methylamidated MethylatedProteinCterm

-membrane

-in:file:spanfile target.span

-membrane:no_interpolate_Mpair

-membrane:Menv_penalties

**1.2 rosetta_cm.xml**

<ROSETTASCRIPTS>

<TASKOPERATIONS>

</TASKOPERATIONS>

<SCOREFXNS>

<ScoreFunction name="stage1" weights="stage1_membrane.wts" symmetric="0">

<Reweight scoretype="atom_pair_constraint" weight="1"/>

</ScoreFunction>

<ScoreFunction name="stage2" weights="stage2_membrane.wts" symmetric="0">

<Reweight scoretype="atom_pair_constraint" weight="0.5"/>

</ScoreFunction>

<ScoreFunction name="fullatom" weights="stage3_rlx_membrane.wts" symmetric="0">

<Reweight scoretype="atom_pair_constraint" weight="0.5"/>

</ScoreFunction>

</SCOREFXNS>

<FILTERS>

</FILTERS>

<MOVERS>

<Hybridize name="hybridize" stage1_scorefxn="stage1" stage2_scorefxn="stage2" fa_scorefxn="fullatom" batch="1" stage1_increase_cycles="1.0" stage2_increase_cycles="1.0" linmin_only="1">

<Fragments three_mers="target_3.frags" nine_mers="target_9.frags"/>

<Template pdb="target_on_template1.pdb" cst_file="AUTO" weight="1.000" />

<Template pdb="target_on_template2.pdb" cst_file="AUTO" weight="1.000" />

<Template pdb="target_on_template3.pdb" cst_file="AUTO" weight="1.000" />

</Hybridize>

</MOVERS>

<APPLY_TO_POSE>

</APPLY_TO_POSE>

<PROTOCOLS>

<Add mover="hybridize"/>

</PROTOCOLS>

</ROSETTASCRIPTS>

**1.3 relax**

-in:fix_disulf target.disulfide

-in:file:spanfile target.span

-membrane:no_interpolate_Mpair

-membrane:Menv_penalties

-score:weights membrane_highres_Menv_smooth.wts

-relax:dualspace

-relax:minimize_bond_angles

-set_weights cart_bonded .5 pro_close 0

-default_max_cycles 200

-out:file:fullatom

-out:pdb

**2. C3 Symmetry**

-in:file:s target_INPUT.pdb

-in:file:native target.pdb

-ignore_unrecognized_res

-symmetry:symmetry_definition symm_def_trimer.dat -symmetry:initialize_rigid_body_dofs

-symmetry:symmetric_rmsd

-packing:ex1

-packing:ex2aro

-out:nstruct 100

-out:file:fullatom

**3. Loop remodel**

**3.1 remodel**

-in:file:s target.pdb

-remodel:blueprint target.remodel

-run:chain A

-remodel:num_trajectory 100

-remodel:quick_and_dirty

-out:path:all output_files

-out:file:scorefile target.sc

**3.2 cartesian relax**

-in:file:s target.pdb

-relax:constrain_relax_to_native_coords

-in:file:native target_4ntw.pdb

-ignore_unrecognized_res

-nstruct 100

-relax:coord_cst_stdev 0.05

-relax:ramp_constraints false

-relax:cartesian

-score:weights ref2015_cart

-relax:min_type lbfgs_armijo_nonmonotone

-relax:script cart.script

-ex1

-ex2

-flip_HNQ

-no_optH false

-repair_sidechains true

-crystal_refine

-constraints:cst_file sc.cst

-constraints:cst_weight 3

-out:path:pdb

-out:file:scorefile target_mp_cart.sc

**3.3 cart.script**

switch:cartesian

repeat 2

ramp_repack_min 0.02 0.01 1.0 50

ramp_repack_min 0.250 0.01 0.5 50

ramp_repack_min 0.550 0.01 0.0 100

ramp_repack_min 1 0.00001 0.0 200

accept_to_best

endrepeat

**4. mp relax before ddG**

**4.1 mp relax**

-parser:protocol membrane_relax.xml

-in:file:s target_mp.pdb

-mp:scoring:hbond

-mp:setup:spanfiles target_mp.span

-nstruct 100

-relax:fast

-relax:jump_move true

-out:path:pdb

-out:file:scorefile target.sc

-packing:pack_missing_sidechains 0

-ignore_unrecognized_res

-ex1

-ex2

-flip_HNQ

-repair_sidechains true

-crystal_refine

**4.2 cartesian relax**

-in:file:s model.pdb

-use_input_sc

-constrain_relax_to_start_coords

-ignore_unrecognized_res

-nstruct 100

-relax:coord_constrain_sidechains

-relax:cartesian

-score:weights ref2015_cart

-relax:min_type lbfgs_armijo_nonmonotone

-relax:script cart.script # refers to 3.3 part

-ex1

-ex2

-flip_HNQ

-no_optH false

-crystal_refine

-out:path:pdb

-out:file:scorefile model_ddg_prep.sc

**4.3 Cartesian_ddG**

-in:file:s target.pdb

-ddg:mut_file XXXX_8A.mutfile

-in_span target.span

-ddg:iterations 10

-ddg::cartesian

-ddg::dump_pdbs true

-ddg:bbnbrs 1

-fa_max_dis 8.0

-score:weights ref2015_cart

-relax:cartesian

-relax:min_type lbfgs_armijo_nonmonotone

-ex1

-ex2

-use_input_sc

-flip_HNQ

-optimization:default_max_cycles 500

-crystal_refine

**4.4 mp scoring**

Rosetta/main/source/bin/score_jd2.macosclangrelease \

-database Rosetta/main/database

-in:file:s target.pdb

-in:membrane

-mp:setup:spanfiles target.span

-score:weights mpframework_smooth_fa_2012.wts
